# Supplementary material for: Mindfulness-based programmes for mental health promotion in adults in nonclinical settings: A systematic review and meta-analysis of randomised controlled trials
Source: PLoS Med. 2021 Jan 11;18(1):e1003481. doi: 10.1371/journal.pmed.1003481 (PMC7799763; doi:10.1371/journal.pmed.1003481)
Supplement: S1 Appendix — (PDF) [file pmed.1003481.s001.pdf]

Mindfulness-Based Programmes for Mental Health Promotion in Adults in Non-clinical Settings: A Systematic Review and Meta-Analysis of Randomised Controlled Trials

Supplementary materials (S1 Appendix)

Contents

Supplementary methods 8

Table 1. Search strategies. 8

Table 2. Pre-piloted extraction forms. 11

Table 3. Definitions to refine primary outcome selection. 12

Supplementary results 13

Secondary outcomes 13

Comparison with passive control groups 13

Comparison with active non-specific control groups 14

Comparison with active specific control groups 14

Risk-of-bias Source-specific Sensitivity Analyses 15

Table 4. Summary of secondary outcome results. 16

Table 5. Conference abstracts for which too much information was missing to assess eligibility. 18

Table 6. Comparison of MBPs with passive control groups: multivariate meta-analysis of anxiety, depression, distress, and wellbeing outcomes at post-intervention, (post-int), 1-6 months follow-up, (1-6m) and 6+ months follow-up (+6m). Exchangeable between-study variance-covariance matrices (0.1). 19

Table 7. Comparison of MBPs with active non-specific control groups: multivariate meta-analysis of anxiety, depression, distress, and wellbeing at post-intervention, (post-int), 1-6 months follow-up, (1-6m) and 6+ months follow-up (+6m). Exchangeable between-study variance-covariance matrices (0.7). 20

Table 8. Comparison of MBPs with active specific control groups: multivariate meta-analysis of anxiety, depression, distress, and wellbeing at post-intervention, (post-int), 1-6 months follow-up, (1-6m) and 6+ months follow-up (+6m). Exchangeable between-study variance-covariance matrices (0.1). 21

Table 9. Comparison of MBPs with passive control groups: multivariate meta-analysis of cognitive functioning at post-intervention, (Post-int), and 1-6 months follow-up (1-6m). Results were robust to the outcome-specific correlation sensitivity analysis. 22

Table 10. Comparison of MBPs with active non-specific control groups: multivariate meta-analysis of cognitive functioning at post-intervention. 23

Table 11. Comparison of MBPs with active specific control groups: univariate meta-analysis of cognitive functioning at post-intervention. 24

Table 12. Comparison of MBPs with passive control groups: multivariate meta-analysis of real-life functioning at post-intervention, (Post-int), 1-6 months follow-up (1-6m), and 6+ months follow-up (+6m). Exchangeable between-study variance-covariance matrices (0.8). 25

Table 13. Comparison of MBPs with active non-specific control groups: multivariate meta-analysis of real-life functioning at post-intervention, (Post-int), and 1-6 months follow-up (1-6m). Exchangeable between-study variance-covariance matrices (0.1). 26

Table 14. Comparison of MBPs with active specific control groups: multivariate meta-analysis of real-life functioning at post-intervention, (Post-int), 1-6 months follow-up (1-6m), and 6+ months follow-up (+6m). Exchangeable between-study variance-covariance matrices (0.9).. 27

Table 15. Comparison of MBPs with passive control groups: multivariate meta-analysis of relationship with the self at post-intervention, (Post-int), and 1-6 months follow-up (1-6m).. 28

Table 16. Comparison of MBPs with active non-specific control groups: multivariate meta-analysis of relationship with the self at post-intervention, (Post-int), and 1-6 months follow-up (1-6m). Exchangeable between-study variance-covariance matrices (0.8). 29

Table 17. Comparison of MBPs with active specific control groups: multivariate meta-analysis of relationship with the self at post-intervention, (Post-int), 1-6 months follow-up (1-6m), and 6+ months follow-up (+6m). Exchangeable between-study variance-covariance matrices (0.5). 30

Table 18. Comparison of MBPs with passive control groups: univariate meta-analysis of psychosomatic symptoms at post-intervention. 31

Table 19. Comparison of MBPs with passive control groups: univariate meta-analysis of psychosomatic symptoms at 1-6 months follow-up. 32

Table 20. Comparison of MBPs with active non-specific control groups: multivariate meta-analysis of psychosomatic symptoms at post-intervention, (Post-int), and 1-6 months follow-up (1-6m). Exchangeable between-study variance-covariance matrices (0.5). 33

Table 21. Comparison of MBPs with active specific control groups: multivariate meta-analysis of psychosomatic symptoms at post-intervention, (post-int), 1-6 months follow-up (1-6m), and 6+ months follow-up (+6m). Exchangeable between-study variance-covariance matrices (0.8). 34

Table 22. Comparison of MBPs with passive control groups: multivariate meta-analysis of dispositional mindfulness at post-intervention, (post-int), 1-6 months follow-up (1-6m), and 6+ months follow-up (+6m). Exchangeable between-study variance-covariance matrices (0.9).. 35

Table 23. Comparison of MBPs with active non-specific control groups: multivariate meta-analysis of dispositional mindfulness at post-intervention, (post-int), and 1-6 months follow-up (1-6m). 36

Table 24. Comparison of MBPs with active specific control groups: multivariate meta-analysis of dispositional mindfulness at post-intervention, (post-int), 1-6 months follow-up (1-6m), and

6+ months follow-up (+6m). Exchangeable between-study variance-covariance matrices (0.9). 37

Table 25. Risk of bias assessment for individual studies. 38

Table 26. Potentially eligible trial registry records with no available results.......................... 42

Table 27. Grading of Recommendations Assessment, Development and Evaluation (GRADE) assessment details by confidence domain: risk of bias, non-reporting bias, imprecision, inconsistency, and indirectness. 43

Table 28. Sensitivity analysis of methodological quality removing high-risk-of-bias trials. Comparison of MBPs with passive control groups: multivariate meta-analysis of anxiety, depression, distress, and wellbeing at post-intervention, (post-int), 1-6 months follow-up, (1-6m) and 6+ months follow-up (+6m). 44

Table 29. Sensitivity analysis of methodological quality removing high-risk-of-bias trials. Comparison of MBPs with active non-specific control groups: multivariate meta-analysis of anxiety, depression, distress, and wellbeing at post-intervention, (post-int), 1-6 months follow-up, (1-6m) and 6+ months follow-up (+6m). 45

Table 30. Sensitivity analysis of methodological quality removing high-risk-of-bias trials. Comparison of MBPs with active specific control groups: multivariate meta-analysis of anxiety, depression, distress, and wellbeing at post-intervention, (post-int), 1-6 months follow-up, (1-6m) and 6+ months follow-up (+6m). 46

Table 31. Sensitivity analysis of methodological quality removing trials at high risk of bias due to deviations from intended interventions. Comparison of MBPs with passive control groups: multivariate meta-analysis of anxiety, depression, distress, and wellbeing at post-intervention, (post-int), 1-6 months follow-up, (1-6m) and 6+ months follow-up (+6m). 47

Table 32. Sensitivity analysis of within-study correlation assumptions using Riley's method. Comparison of MBPs with passive control groups: multivariate meta-analysis of anxiety, depression, distress, and wellbeing at post-intervention, (post-int), 1-6 months follow-up, (1-6m) and 6+ months follow-up (+6m). 48

Table 33. Sensitivity analysis of within-study correlation assumptions using Riley's method. Comparison of MBPs with active non-specific control groups: multivariate meta-analysis of

anxiety, depression, distress, and wellbeing at post-intervention, (post-int), 1-6 months follow-up, (1-6m) and 6+ months follow-up (+6m). 49

Table 34. Sensitivity analysis of within-study correlation assumptions using Riley's method. Comparison of MBPs with active specific control groups: multivariate meta-analysis of anxiety, depression, distress, and wellbeing at post-intervention, (post-int), 1-6 months follow-up, (1-6m) and 6+ months follow-up (+6m). 50

Table 35. Sensitivity analysis of within study correlation assumptions. Univariate meta-analyses of anxiety, depression, distress and wellbeing outcomes comparing MBPs with passive control groups. 51

Table 36. Sensitivity analysis of within study correlation assumptions. Univariate meta-analyses of anxiety, depression, distress and wellbeing outcomes compared with active non-specific control groups. 54

Table 37. Sensitivity analysis of within study correlation assumptions. Univariate meta-analyses of anxiety, depression, distress and wellbeing outcomes comparing MBPs with active specific control groups. 56

Table 38. Sensitivity analysis excluding data from samples N<30. Comparison of MBPs with passive control groups: multivariate meta-analysis of anxiety, depression, distress, and wellbeing at post-intervention, (post-int), 1-6 months follow-up, (1-6m) and 6+ months follow-up (+6m). The prediction intervals are shown in the second table. 58

Table 39. Sensitivity analysis setting estimate to +1 SE. Comparison of MBPs with passive control groups: multivariate meta-analysis of anxiety, depression, distress, and wellbeing at post-intervention, (post-int), 1-6 months follow-up, (1-6m) and 6+ months follow-up (+6m). 59

Table 40. Sensitivity analysis setting estimate to +1 SE. Comparison of MBPs with active non-specific control groups: multivariate meta-analysis of anxiety, depression, distress, and wellbeing at post-intervention (post-int), 1-6 months follow-up, (1-6m) and 6+ months follow-up (+6m). 60

Table 41. Sensitivity analysis setting estimate to +1 SE. Comparison of MBPs with active specific control groups: multivariate meta-analysis of anxiety, depression, distress, and

wellbeing at post-intervention, (post-int), 1-6 months follow-up, (1-6m) and 6+ months follow-up (+6m). 61

Table 42. Sensitivity analysis setting estimate to -1 SE. Comparison of MBPs with passive control groups: multivariate meta-analysis of anxiety, depression, distress, and wellbeing at post-intervention, (post-int), 1-6 months follow-up, (1-6m) and 6+ months follow-up (+6m). The prediction intervals are shown in the second table. 62

Table 43. Sensitivity analysis setting estimate to -1 SE. Comparison of MBPs with active non-specific control groups: multivariate meta-analysis of anxiety, depression, distress, and wellbeing at post-intervention, (post-int), 1-6 months follow-up, (1-6m) and 6+ months follow-up (+6m). The prediction intervals are shown in the second table. 63

Table 44. Sensitivity analysis setting estimate to -1 SE. Comparison of MBPs with active specific control groups: multivariate meta-analysis of anxiety, depression, distress, and wellbeing at post-intervention, (post-int), 1-6 months follow-up, (1-6m) and 6+ months follow-up (+6m). 64

Table 45 Sensitivity analysis excluding studies with unclear teacher competence. Comparison of MBPs with passive control groups: multivariate meta-analysis of anxiety, depression, distress, and wellbeing at post-intervention, (post-int), 1-6 months follow-up, (1-6m) and 6+ months follow-up (+6m). The prediction intervals are shown in the second table. 65

Table 46. Sensitivity analysis excluding studies with unclear teacher competence. Comparison of MBPs with active non-specific control groups: multivariate meta-analysis of anxiety, depression, distress, and wellbeing at post-intervention, (post-int), 1-6 months follow-up, (1-6m) and 6+ months follow-up (+6m). 66

Table 47. Sensitivity analysis excluding studies with unclear teacher competence. Comparison of MBPs with active specific control groups: multivariate meta-analysis of anxiety, depression, distress, and wellbeing at post-intervention, (post-int), 1-6 months follow-up, (1-6m) and 6+ months follow-up (+6m). 67

Table 48. Multivariate Meta-regression. Comparison of MBPs with passive control groups, outcomes at 1-6 months follow-up. USA studies= studies from the USA; selective MBP=selective intervention; indicated MBP=indicated intervention; Psychoeducation= adding

psychoeducation component and/or non-meditative psychological exercises; Physical exercise= adding physical exercise component; Arts= adding arts component. 68

Table 49. Meta-regression. Comparison of MBPs with active specific control groups, outcome distress at 1-6 months follow-up. Selective MBP=selective intervention; indicated MBP=indicated intervention; Psychoeducation= adding psychoeducation component and/or non-meditative psychological exercises; Physical exercise= adding physical exercise component; Other meditation= adding other types meditation component; Arts= adding arts component. Psychoeducation and other meditation are omitted because of collinearity. 69

Fig 1. Funnel plot for univariate meta-analysis for the depression outcome at 1-6 months post-intervention for MBPs compared with passive controls. 70

Fig 2. Funnel plot for univariate meta-analysis distress 1-6 months post-intervention compared with passive controls. 71

Fig 3. Funnel plot for univariate meta-analysis distress 1-6 months post-intervention compared with active specific controls. 72

Supplementary methods

Table 1. Search strategies.

Database	Strategy
Allied and Complementary Medicine (AMED) (through OVID)	S1: meditation/<br>S2: mindful*.mp.<br>S3: meditat*.mp.<br>S4: clinical trials/ or randomized controlled trials/ or double blind method/ or random allocation/<br>S5: RCT.mp.<br>S6: (random* adj1 allocat*).mp.<br>S7: (random* adj1 assign*).mp.<br>S8: randomis*.mp.<br>S9: randomiz*.mp.<br>S10: 1 or 2 or 3<br>S11: 4 or 5 or 6 or 7 or 8 or 9<br>S12: 10 and 11
Applied Social Sciences Index and Abstracts (ASSIA)	S1: ab((mindful* OR meditat*) AND (randomise* OR randomize* OR RCT OR "random allocation" OR "random assignment")) OR ti((mindful* OR meditat*) AND (randomise* OR randomize* OR RCT OR "random allocation" OR "random assignment"))
Cochrane Central Register of Controlled Trials (CENTRAL)	S1: MH “Mindfulness”<br>S2: AB (mindfulness or mindfulness or meditat*) or TI (mindfulness or mindfulness or meditat*)<br>1 or 2
Cumulative Index to Nursing and Allied Health Literature (CINAHL) (through EBSCO)	S1: (MH "Mindfulness+")<br>S2: (MH "Mindfulness")<br>S3: (MH "Meditation")<br>S4: TI (mindful* OR meditat*) OR AB (mindful* OR meditat*)<br>S5: S1 OR S2 or S3 or S4<br>S6: MH "Clinical Trials+"<br>S7: PT Clinical trial<br>S8: TX clinic* n1 trial*<br>S9: TX ((singl* n1 blind*) or (singl* n1 mask*)) or TX ((doubl* n1 blind*) or (doubl* n1 mask*)) or TX ((tripl* n1 blind*) or (tripl* n1 mask*)) or TX ((trebl* n1 blind*) or (trebl* n1 mask*))<br>S10: TX randomi* control* trial*<br>S11: (MH "Random Assignment")<br>S12: TX random* allocat*<br>S13: TX placebo*<br>S14: (MH "Placebos")<br>S15: (MH "Quantitative Studies")<br>S16: TX allocat* random*<br>S17: S6 OR S7 OR S8 OR S9 OR S10 OR S11 OR S12 OR S13 OR S14 OR S15 OR S16<br>S18: S5 AND S17
Excerpta Medica Database (EMBASE) (through OVID)	S1 exp meditation/ or exp mindfulness/<br>S2 (mindfullness or mindfulness or meditat*).ab. or (mindfullness or mindfulness or meditat*).ti.<br>S3 1 or 2<br>S4 clinical trial/<br>S5 randomized controlled trial/<br>S6 controlled clinical trial/<br>S7 multicenter study/<br>S8 phase 3 clinical trial/<br>S9 phase 4 clinical trial/

	S10 double blind procedure/<br>S11 placebo/<br>S12 exp randomization/<br>S13 (randomi?ed controlled trial$ or rct or (random$ adj2 allocat$) or single blind$ or double blind$ or ((treble or triple) adj blind$) or placebo$).tw.<br>S14 Prospective Study.mp. or prospective study/<br>S15 4 or 5 or 6 or 7 or 8 or 9 or 10 or 11 or 12 or 13 or 14<br>S16 3 and 15
Education Resources Information Center (ERIC) (through EBSCO)	S1: AB (meditat* OR mindful*) OR TI (meditat* OR mindful*)<br>S2: randomis* OR randomiz* OR RCT OR "random* allocat*" OR "random* assign*"<br>S3: S1 AND S2
Electronic Theses Online Service (EThOS)	S1: Advanced search: mindful (title) AND randomise (any word)
Medical Literature Analysis and Retrieval System Online (MEDLINE) (through OVID)	S1: Randomized Controlled Trials as Topic/<br>S2: randomized controlled trial/<br>S3: Random Allocation/<br>S4: Double Blind Method/<br>S5: Single Blind Method/<br>S6: clinical trial/<br>S7: clinical trial, phase i.pt<br>S8: clinical trial, phase ii.pt<br>S9: clinical trial, phase iii.pt<br>S10: clinical trial, phase iv.pt<br>S11: controlled clinical trial.pt<br>S12: randomized controlled trial.pt<br>S13: multicenter study.pt<br>S14: clinical trial.pt<br>S15: exp Clinical Trials as topic/<br>S16: or/1-15<br>S17: (clinical adj trial$).tw<br>S18: ((singl$ or doubl$ or treb$ or tripl$) adj (blind$3 or mask$3)).tw<br>S19: PLACEBOS/<br>S20: placebo$.tw<br>S21: randomly allocated.tw<br>S22: (allocated adj2 random$).tw<br>S23: or/17-22<br>S24: 16 or 23<br>S25: case report.tw<br>S26: letter/<br>S27: historical article/<br>S28: or/25-27<br>S29: 24 not 28<br>S30: exp meditation/ or exp mindfulness/<br>S31: (mindfulness or mindfulness or meditat*).ab or (mindfulness or mindfulness or meditat*).ti<br>S32: 30 or 31<br>S33: 32 and 29
ProQuest	S1: ab((mindful* OR meditat*) AND (randomise* OR randomize* OR RCT OR "random allocation" OR "random assignment")) OR ti((mindful* OR meditat*) AND (randomise* OR randomize* OR RCT OR "random allocation" OR "random assignment"))
PsycINFO (through EBSCO)	S1: DE "Meditation" OR DE "Mindfulness"<br>S2: AB (mindfullness OR mindfulness OR meditat* OR mindful*) OR TI (mindfullness OR mindfulness OR meditat* OR mindful*)<br>S3: S1 OR S2<br>S4: AB ((randomized controlled trial) OR (Random Allocation) OR (Double Blind Method) OR (Single Blind Method) OR (clinical trial) OR (clinical trial, phase i.pt) OR

	(clinical trial, phase ii.pt) OR (clinical trial, phase iii.pt) OR (clinical trial, phase iv.pt) OR (controlled clinical trial.pt) OR (randomized controlled trial.pt) OR (multicenter study.pt) OR (clinical trial.pt) OR ((clinical adj trial$).tw) OR (((singl$ or doubl$ or treb$ or tripl$) adj (blind$3 or mask$3)).tw) OR PLACEBOS OR (placebo$.tw) OR (randomly allocated.tw) OR ((allocated adj2 random$).tw)) OR TI ((randomized controlled trial) OR (Random Allocation) OR (Double Blind Method) OR (Single Blind Method) OR (clinical trial) OR (clinical trial, phase i.pt) OR (clinical trial, phase ii.pt) OR (clinical trial, phase iii.pt) OR (clinical trial, phase iv.pt) OR (controlled clinical trial.pt) OR (randomized controlled trial.pt) OR (multicenter study.pt) OR (clinical trial.pt) OR ((clinical adj trial$).tw) OR (((singl$ or doubl$ or treb$ or tripl$) adj (blind$3 or mask$3)).tw) OR PLACEBOS OR (placebo$.tw) OR (randomly allocated.tw) OR ((allocated adj2 random$).tw))<br>S5: S3 AND S4
Scopus	S1: (mindful* OR meditat*) AND (randomise* OR randomize* OR RCT OR "random allocation" OR "random assignment")
Web of Science	S1: advanced search: TS=((mindful* OR meditat*) AND (randomise* OR randomize* OR RCT OR "random allocation" OR "random assignment"))
World Health Organization (WHO) International Clinical Trials Registry Platform (ICTRP)	S1: mindful or meditat

Table 2. Pre-piloted extraction forms.

Form Tab	Information collected
Study Identification	Sponsorship sources, conflicts of interest, country, study setting, corresponding authors, institution, emails, date recruitment started, and year first published.
Methods	Study design, conceptualisation of mindfulness, incentives for participants, number of participants (total randomised and per arm), and power calculation.
Population	Inclusion and exclusion criteria, type of participant, group differences, baseline characteristics
Interventions	Intervention name, mention of intervention manual, whether it was an adaptation of another intervention (rationale), intensity, mindfulness components (type, frequency and duration), non-mindfulness components (type, frequency and duration), home practice length and type, group size, any individual tailoring, any booster sessions or support after the end of the programme, adherence to intervention manual, intervention setting, teacher competence, teacher characteristics, response to intervention (attendance, satisfaction, reasons for missing sessions), and whether participants paid to do the course.
Outcomes	Outcome measure used, time points, group sizes, effect measures available and extracted effect sizes.

Table 3. Definitions to refine primary outcome selection.

Primary outcome	Requirement
Distress	has to measure more than one negative emotion
Wellbeing	has to be more than one positive emotion
Anxiety	has to include physical symptoms and functioning impairment.
Depression	has to include general anhedonia, worthlessness, physical symptoms and functioning

Supplementary results

Secondary outcomes

Table 4 contains a summary of secondary outcome results, which are summarised below. The systematic review search date for the secondary outcomes was January 2020.

Comparison with passive control groups

At post-intervention (measured within one month of completing the intervention), in comparison with passive control groups, on average MBPs improved anxiety, depression, psychological distress, and mental wellbeing, (Table 6). The prediction intervals indicated that post-intervention anxiety will be reduced following MBPs in more than 95% of the scenarios, but improvement is not homogeneous for the rest of the outcome domains. There was no evidence that improvements following MBPs in depression, distress or wellbeing remained six or more months post-intervention (no studies for anxiety). However, only one study measured depression, four distress and three wellbeing at six or more months post-intervention, so results need to be interpreted with caution.

There is evidence for a modest and heterogeneous improvement in cognitive functioning following MBPs compared to passive controls shortly after intervention completion, with no significant differences at one-to-six-months follow-up (Table 9). MBPs improved real-life functioning at post-intervention in comparison with passive controls (small effect, Table 12). Effects may last for up to six months, with no reliable evidence on longer effects. MBPs improved the relationship with the self for up to at least six months (Table 15, no data on longer-term outcomes), and dispositional mindfulness for longer (Table 22). MBPs reduced psychosomatic symptoms shortly

after course completion (Table 18), but no evidence supported effects persisting for any longer (Table 19). None of these effects was generalisable across settings.

Comparison with active non-specific control groups

In comparison with active non-specific control groups at post-intervention, results supported improvements following MBPs in anxiety, depression, distress and wellbeing. However, reliability is low due to a mix of few studies contributing data, borderline p values, and prediction intervals that included adverse scenarios (Table 7). No studies measured these outcomes six or more months after the interventions. We found no evidence for improvements following MBPs in cognitive function (only three studies measuring this, Table 10), real-life functioning (only four studies measuring it, Table 13), psychosomatic outcomes (only 2 studies measuring it, Table 20), or dispositional mindfulness (Table 23). MBPs improved the relationship with the self for up to at least six months (Table 16, no data on longer-term outcomes). However, this effect is not generalisable to all implementation settings.

Comparison with active specific control groups

Compared with active specific control groups, findings supported a modest superiority of MBPs in improving depression and wellbeing, but not distress and anxiety, at post-intervention (Table 8). Prediction intervals included null or unfavourable effects. Very few studies measured these outcomes six or more months after the intervention, with no significant differences between groups. We found no evidence for improvement in cognitive functioning (Table 11), real-life functioning (Table 14), relationship with the self (Table 17), psychosomatic symptoms (Table 21), or dispositional mindfulness (Table 24).

Risk-of-bias Source-specific Sensitivity Analyses

Source-specific sensitivity analyses could be conducted for risk-of-bias sources of randomisation, deviations from intended interventions, and missing outcome data; there was not enough risk variance for the other sources to meaningfully remove higher-risk trials. Source-specific analyses gave similar results to the overall-risk sensitivity analyses, except that in the comparison with passive controls the effects of MBPs on depression, distress and wellbeing remained significant and with narrower prediction intervals after removing trials at high risk of bias due to deviations from the intended interventions (a bias that tended to dilute intervention effects due to contamination between arms, Table 31).

Table 4. Summary of secondary outcome results.

Control group	Outcome domain	Time point	n of trials	SMD (95%CI) p	95%PI	Missed trials*
Passive	Anxiety	P-int	19	-0.70 (-0.85, -0.54), <0.001	-1.29, -0.10	0
	Depression	P-int	37	-0.45 (-0.57, -0.33), <0.001	-1.04, 0.14	1 (3%)
	Depression	6+m	1	-0.19 (-0.78, 0.40), 0.53	-1.02, 0.64	0
	Distress	P-int	61	-0.45 (-0.54, -0.36), <0.001	-1.03, 0.14	4 (6%)
	Distress	6+m	4	-0.16 (-0.48, 0.15), 0.31	-0.82, 0.50	0
	Wellbeing	P-int	25	0.34 (0.21 0.48), <0.001	-0.25, 0.94	3 (11%)
	Wellbeing	6+m	3	0.25 (-0.10, 0.60), 0.17	-0.43, 0.93	1 (25%)
	Cog. Func.	P-int	13	0.25 (0.06, 0.44) , 0.009	-0.32, 0.83	1 (7%)
	Cog. Func.	1-6m	2	0.03 (-0.52, 0.58) , 0.91	-1.04, 1.10	1 (33%)
	Real Func.	P-int	25	0.27 (0.12, 0.43), <0.001	-0.38, 0.93	1 (4%)
	Real Func.	1-6m	14	0.23 (0.05, 0.40), 0.013	-0.44, 0.89	1 (7%)
	Real Func.	6+m	1	0.09 (-0.56, 0.74), 0.79	-0.85, 1.02	1 (50%)
	Rel. Self	P-int	20	0.77 (0.47, 1.07), <0.001	-0.60, 2.13	2 (10%)
	Rel. Self	1-6m	8	0.71 (0.41, 1.02), <0.001	-0.38, 1.80	0
	Psychosom.	P-int	14	-0.41 (-0.58, -0.24), <0.001	-0.94, 0.12	1 (7%)
	Psychosom.	1-6m	7	-0.25 (-0.65, 0.16), 0.19	-1.27, 0.78	1 (13%)
	Mindfulness	P-int	50	0.54 (0.41, 0.67), <0.001	-0.35, 1.42	5 (9%)
	Mindfulness	1-6m	18	0.56 (0.40, 0.72), <0.001	-0.33, 1.45	2 (10%)
	Mindfulness	6+m	2	0.52 (0.24. 0.80), <0.001	-0.40, 1.44	1 (33%)
Active non-specific	Anxiety	P-int	4	-0.55 (-0.95, -0.15), 0.007	-1.69, 0.58	1 (20%)
	Depression	P-int	7	-0.43 (-0.77, -0.08), 0.016	-1.53, 0.68	0
	Distress	P-int	9	-0.38 (-0.70, -0.06), 0.021	-1.48, 0.72	1 (10%)
	Wellbeing	P-int	1	3.00 (1.70, 4.30), <0.001	1.18, 4.83	0
	Cog. Func.	P-int	3	0.08 (-0.66, 0.81), 0.84	-8.82, 8.98	0
	Real Func.	P-int	3	0.04 (-0.75, 0.83), 0.92	-3.18, 3.26	0
	Real Func.	1-6m	2	0.12 (-0.87, 1.12), 0.81	-3.36, 3.60	0
	Rel. Self	P-int	3	0.80 (0.26, 1.34), 0.004	-1.49, 3.09	0
	Rel. Self	1-6m	3	0.73 (0.18, 1.28), 0.010	-1.58, 3.03	0
	Psychosom.	P-int	2	-0.36 (-0.74, 0.03), 0.067	NA	0
	Psychosom.	1-6m	1	-0.29 (-0.89, 0.30), 0.34	NA	0
	Mindfulness	P-int	7	0.21 (-0.02, 0.44), 0.068	-0.42, 0.85	0
	Mindfulness	1-6m	6	0.28 (-0.06, 0.61), 0.11	-0.44, 0.99	0
Active specific	Anxiety	P-int	7	-0.12 (-0.27, 0.04), 0.14	-0.45, 0.21	1 (13%)
	Depression	P-int	17	-0.24 (-0.36, -0.13), <0.001	-0.55, 0.07	0
	Depression	6+m	3	-0.05 (-0.33, 0.24), 0.75	-0.46, 0.37	0
	Distress	P-int	24	-0.07 (-0.18, 0.03), 0.15	-0.38, 0.23	5 (17%)

Distress	6+m	4	-0.03 (-0.27, 0.22), 0.84	-0.41, 0.36	0
Wellbeing	P-int	10	0.17 (0.03, 0.31), 0.015	-0.15, 0.49	3 (23%)
Wellbeing	6+m	1	-0.00 (-0.36, 0.36), 0.99	-0.48, 0.47	1 (50%)
Cog. Func.	P-int	7	-0.03 (-0.29, 0.24), 0.80	-0.47, 0.41	0
Real Func.	P-int	6	0.03 (-0.17, 0.23), 0.76	-0.21, 0.27	1 (14%)
Real Func.	1-6m	4	0.01 (-0.21, 0.23), 0.93	-0.26, 0.28	1 (20%)
Real Func.	6+m	3	0.02 (-0.18, 0.23), 0.82	-0.22, 0.27	0
Rel. Self	P-int	6	0.13 (-0.15, 0.41), 0.37	-0.53, 0.78	3 (33%)
Rel. Self	1-6m	1	0.40 (-0.12, 0.92), 0.13	-0.47, 1.27	1 (50%)
Rel. Self	6+m	1	0 .00 (-0.90, 0.90), 1.00	-1.30, 1.30	1 (50%)
Psychosom.	P-int	7	-0.18 (-0.38, 0.02), 0.068	-0.72, 0.36	1 (13%)
Psychosom.	1-6m	4	-0.13 (-0.36, 0.09), 0.25	-0.70, 0.43	2 (33%)
Psychosom.	6+m	2	-0.15 (-0.42, 0.13), 0.29	-0.74, 0.45	0
Mindfulness	P-int	19	0.14 (-0.02, 0.29), 0.09	-0.49, 0.76	2 (10%)
Mindfulness	1-6m	7	0.12 (-0.07, 0.31), 0.20	-0.52, 0.76	1 (13%)
Mindfulness	6+m	3	0.15 (-0.09, 0.40), 0.21	-0.51, 0.81	1 (25%)

* Number of trials with non-reported data for the corresponding outcome. Abbreviations: 1-6m=1 to 6 months post-intervention follow up, 6+ months= more than 6 months post intervention follow up, CI= confidence interval for overall mean, Cog. Func = Cognitive functioning, n=number, P-int=post-intervention, PI= prediction interval for new study, Psychosom = Psychosomatic outcomes, Real func = Real life functioning, Rel. Self = Relationship with self, SMD=standardised mean difference.

Table 5. Conference abstracts for which too much information was missing to assess eligibility.

1	Siwik, C., Phillips, K., Salmon, P., Litvan, I., Filoteo, V., Rebholz, W., ... & van der Gryp, K. (2018, April). An MBSR intervention for parkinson's disease patients and caregiving partners: Effects on distress, social support, cortisol, and inflammation. In Psychosomatic Medicine (Vol. 80, No. 3, pp. A128-A128). Two Commerce SQ, 2001 Market St, Philadelphia, PA 19103 USA: Lippincott Williams & Wilkins.
2	Greven, C.; Bogels, S.; Dammers, J.; Buitelaar, J.; Speckens, A. Mindfulness for children with ADHD and Mindful Parenting (MindChamp): a randomised controlled trial. Journal of Neural Transmission Nov 2019;126(11):1568-1569 2019 Nov
3	Orosa Duarte, A.; Mediavilla, R.; Lopez Herrero, V.; Garde Gonzalez, J.; Rodriguez Vega, B.; Munoz San Jose, A.; Palao Tarrero, A.; Bravo Ortiz, M. F.; Bayon Perez, C. Mindfulness-based intervention through a smartphone application versus mindfulness-based stress reduction (MBSR) program in healthcare students: a randomised controlled trial. European Psychiatry Apr 2019;56():S569-S569 2019 Apr
4	Exploring effects of aerobic exercise and mindfulness training on cognitive function in older adults at risk of dementia: The active minds study. Circulation 2018;138(Supplement 1): Netherlands Lippincott Williams and Wilkins 2018
5	Wang, Z. Y.; Jin, Z. The Effects of mindfulness-based cognitive therapy (MBCT) on anxiety and depression among professional women: Increased EEG gamma and alpha brainwave amplitude. Basic and Clinical Pharmacology and Toxicology 2018;123(Supplement 3):103 Netherlands Blackwell Publishing Ltd 2018
6	Kiseleva, N.; Kiselev, S. Mindfulness training can reduce prenatal maternal stress. Journal of the Neurological Sciences 2019;405(Supplement):32 Netherlands Elsevier B.V. 2019
7	Kiselev, S.; Volik, I. Influence of mindfulness training on stress reduction during pregnancy. European Psychiatry 2018;48(Supplement 1):S257-S258 Netherlands Elsevier Masson SAS 2018

Table 6. Comparison of MBPs with passive control groups: multivariate meta-analysis of anxiety, depression, distress, and wellbeing outcomes at post-intervention, (post-int), 1-6 months follow-up, (1-6m) and 6+ months follow-up (+6m). Exchangeable between-study variance-covariance matrices (0.1).

```
Multivariate meta-analysis
Variance-covariance matrix = proportional .9*I(11)+.1*J(11,11,1)
Method = reml                              Number of dimensions    =    11
Restricted log likelihood = -119.78835     Number of observations  =    78
```

	Coef.	Std. Err.	z	P>|z|	[95% Conf. Interval]	
Overall mean						
Post-int anxiety	-.694	.081	-8.54	0.000	-.854	-.535
1-6m anxiety	-.563	.120	-4.68	0.000	-.799	-.327
Post-int depression	-.450	.061	-7.32	0.000	-.571	-.330
1-6m depression	-.533	.096	-5.53	0.000	-.722	-.344
+6m depression	-.190	.301	-0.63	0.526	-.780	.399
Post-int distress	-.446	.046	-9.62	0.000	-.537	-.355
1-6m distress	-.447	.068	-6.54	0.000	-.581	-.313
+6m distress	-.163	.162	-1.01	0.314	-.480	.154
Post-int wellbeing	.343	.069	4.94	0.000	.206	.479
1-6m wellbeing	.325	.110	2.94	0.003	.108	.542
+6m wellbeing	.248	.179	1.39	0.165	-.102	.599

Table 7. Comparison of MBPs with active non-specific control groups: multivariate meta-analysis of anxiety, depression, distress, and wellbeing at post-intervention, (post-int), 1-6 months follow-up, (1-6m) and 6+ months follow-up (+6m). Exchangeable between-study variance-covariance matrices (0.7).

```
Multivariate meta-analysis
Variance-covariance matrix = proportional .3*I(8)+.7*J(8,8,1)
Method = reml                            Number of dimensions    =     8
Restricted log likelihood = -23.495745   Number of observations  =    11
```

	Coef.	Std. Err.	z	P>|z|	[95% Conf.	Interval]
Overall mean						
Post-int anxiety	-.552	.204	-2.70	0.007	-.953	-.151
1-6m anxiety	-.474	.201	-2.35	0.019	-.869	-.079
Post-int depression	-.426	.176	-2.42	0.016	-.772	-.080
1-6m depression	-.454	.180	-2.52	0.012	-.808	-.101
Post-int distress	-.378	.164	-2.30	0.021	-.700	-.056
1-6m distress	-.137	.188	-0.73	0.465	-.506	.231
Post-int wellbeing	3.006	.663	4.53	0.000	1.705	4.306
1-6m wellbeing	1.403	.537	2.61	0.009	.350	2.456

Table 8. Comparison of MBPs with active specific control groups: multivariate meta-analysis of anxiety, depression, distress, and wellbeing at post-intervention, (post-int), 1-6 months follow-up, (1-6m) and 6+ months follow-up (+6m). Exchangeable between-study variance-covariance matrices (0.1).

```
Multivariate meta-analysis
Variance-covariance matrix = proportional .9*I(11)+.1*J(11,11,1)
Method = reml                               Number of dimensions    =    11
Restricted log likelihood = -15.552314      Number of observations  =    31
```

	Coef.	Std. Err.	z	P>|z|	[95% Conf.	Interval]
Overall mean						
Post-int anxiety	-.116	.079	-1.46	0.143	-.272	.039
1-6m anxiety	.072	.140	0.51	0.607	-.203	.348
Post-int depression	-.241	.059	-4.06	0.000	-.358	-.125
1-6m depression	-.165	.078	-2.10	0.036	-.319	-.010
+6m depression	-.045	.143	-0.32	0.752	-.326	.235
Post-int distress	-.074	.051	-1.45	0.146	-.175	.026
1-6m distress	-.008	.073	-0.12	0.904	-.151	.134
+6m distress	-.026	.126	-0.21	0.836	-.274	.222
Post-int wellbeing	.169	.069	2.43	0.015	.032	.306
1-6m wellbeing	.028	.108	0.26	0.791	-.183	.240
+6m wellbeing	-.001	.183	-0.01	0.992	-.362	.358

Table 9. Comparison of MBPs with passive control groups: multivariate meta-analysis of cognitive functioning at post-intervention, (Post-int), and 1-6 months follow-up (1-6m). Results were robust to the outcome-specific correlation sensitivity analysis.

```
Multivariate meta-analysis
Variance-covariance matrix = unstructured
Method = reml                                Number of dimensions    =     2
Restricted log likelihood = -5.8048477       Number of observations  =    13
```

	Coef.	Std. Err.	z	P>|z|	[95% Conf. Interval]	
Overall mean						
Post-int	.250	.095	2.61	0.009	.062	.438
1-6m	.030	.281	0.11	0.914	-.520	.581

Table 10. Comparison of MBPs with active non-specific control groups: multivariate meta-analysis of cognitive functioning at post-intervention.

```
 Multivariate meta-analysis
Variance-covariance matrix = unstructured
Method = reml                               Number of dimensions    =     1
Restricted log likelihood = -2.5910786      Number of observations  =     3
```

	Coef.	Std. Err.	z	P>|z|	[95% Conf. Interval]	
Overall mean						
Post-int	.075	.376	0.20	0.842	-.663	.813

Table 11. Comparison of MBPs with active specific control groups: univariate meta-analysis of cognitive functioning at post-intervention.

```
Meta-analysis summary                     Number of studies =      7
Random-effects model                      Heterogeneity:
Method: REML                                          tau2 =  0.0178
SE adjustment: Truncated Knapp-Hartung              I2 (%) =   30.97
                                                        H2 =    1.45

--------------------------------------------------------------------
            Study |    Effect Size    [95% Conf. Interval]  % Weight
------------------+-------------------------------------------------
       Isbel 2019 |         -0.297      -0.739       0.146     12.78
     Lebares 2019 |          0.174      -0.477       0.824      6.87
          Ma 2019 |          0.169      -0.153       0.491     19.64
     MacCoon 2012 |          0.256      -0.266       0.779      9.91
  Malinowski 2017 |         -0.772      -1.387      -0.158      7.58
        Oken 2010 |         -0.007      -0.160       0.147     36.75
       Smart 2017 |          0.000      -0.673       0.673      6.48
------------------+-------------------------------------------------
            theta |         -0.028      -0.292       0.236
--------------------------------------------------------------------
95% prediction interval for theta: [-0.469, 0.413]

Test of theta = 0: t(6) = -0.26                  Prob > |t| = 0.8017
Test of homogeneity: Q = chi2(6) = 10.01           Prob > Q = 0.1241
```

Table 12. Comparison of MBPs with passive control groups: multivariate meta-analysis of real-life functioning at post-intervention, (Post-int), 1-6 months follow-up (1-6m), and 6+ months follow-up (+6m). Exchangeable between-study variance-covariance matrices (0.8).

```
Multivariate meta-analysis
Variance-covariance matrix = proportional .2*I(3)+.8*J(3,3,1)
Method = reml                                Number of dimensions    =     3
Restricted log likelihood = -18.124991       Number of observations  =    29
```

	Coef.	Std. Err.	z	P>|z|	[95% Conf.	Interval]
Overall mean						
Post-int	.272	.078	3.49	0.000	.119	.425
1-6m	.225	.091	2.47	0.013	.046	.404
+6m	.089	.333	0.27	0.789	-.563	.741

Table 13. Comparison of MBPs with active non-specific control groups: multivariate meta-analysis of real-life functioning at post-intervention, (Post-int), and 1-6 months follow-up (1-6m). Exchangeable between-study variance-covariance matrices (0.1).

```
Multivariate meta-analysis
Variance-covariance matrix = proportional .9*I(2)+.1*J(2,2,1)
Method = reml                                Number of dimensions    =     2
Restricted log likelihood = -4.1010413       Number of observations  =     4
```

	Coef.	Std. Err.	z	P>|z|	[95% Conf. Interval]	
Overall mean						
Post-int	.038	.404	0.10	0.924	-.753	.831
1-6m	.122	.507	0.24	0.810	-.871	1.11

Table 14. Comparison of MBPs with active specific control groups: multivariate meta-analysis of real-life functioning at post-intervention, (Post-int), 1-6 months follow-up (1-6m), and 6+ months follow-up (+6m). Exchangeable between-study variance-covariance matrices (0.9).

```
Multivariate meta-analysis
Variance-covariance matrix = proportional .1*I(3)+.9*J(3,3,1)
Method = reml                                Number of dimensions    =     3
Restricted log likelihood = .19744653        Number of observations  =     9
```

	Coef.	Std. Err.	z	P>|z|	[95% Conf. Interval]	
Overall mean						
Post-int	.030	.100	0.30	0.764	-.166	.227
1-6m	.008	.113	0.08	0.939	-.214	.231
+6m	.024	.103	0.23	0.815	-.177	.226

Table 15. Comparison of MBPs with passive control groups: multivariate meta-analysis of relationship with the self at post-intervention, (Post-int), and 1-6 months follow-up (1-6m).

```
Multivariate meta-analysis
Variance-covariance matrix = unstructured
Method = reml                                 Number of dimensions    =     2
Restricted log likelihood = -20.512327        Number of observations  =    21
```

	Coef.	Std. Err.	z	P>|z|	[95% Conf. Interval]	
Overall mean						
Post-int	.765	.153	5.00	0.000	.465	1.065
1-6m	.714	.153	4.66	0.000	.413	1.015

Table 16. Comparison of MBPs with active non-specific control groups: multivariate meta-analysis of relationship with the self at post-intervention, (Post-int), and 1-6 months follow-up (1-6m). Exchangeable between-study variance-covariance matrices (0.8).

```
Multivariate meta-analysis
Variance-covariance matrix = proportional .2*I(2)+.8*J(2,2,1)
Method = reml                              Number of dimensions    =     2
Restricted log likelihood = -3.6406978     Number of observations  =     4
```

	Coef.	Std. Err.	z	P>|z|	[95% Conf. Interval]	
Overall mean						
Post-int	.801	.277	2.89	0.004	.258	1.344
1-6m	.729	.281	2.59	0.010	.176	1.281

Table 17. Comparison of MBPs with active specific control groups: multivariate meta-analysis of relationship with the self at post-intervention, (Post-int), 1-6 months follow-up (1-6m), and 6+ months follow-up (+6m). Exchangeable between-study variance-covariance matrices (0.5).

```
Multivariate meta-analysis
Variance-covariance matrix = proportional .5*I(3)+.5*J(3,3,1)
Method = reml                                  Number of dimensions    =     3
Restricted log likelihood = -2.503334          Number of observations  =     7
```

	Coef.	Std. Err.	z	P>|z|	[95% Conf. Interval]	
Overall mean						
Post-int	.129	.143	0.90	0.366	-.151	.411
1-6m	.398	.265	1.50	0.134	-.122	.919
+6m	0.00	.460	0.00	1.000	-.902	.902

Table 18. Comparison of MBPs with passive control groups: univariate meta-analysis of psychosomatic symptoms at post-intervention.

```
Meta-analysis summary                     Number of studies =     14
Random-effects model                      Heterogeneity:
Method: REML                                          tau2 =  0.0522
SE adjustment: Truncated Knapp-Hartung              I2 (%) =   61.93
                                                        H2 =    2.63

--------------------------------------------------------------------
            Study |    Effect Size    [95% Conf. Interval]  % Weight
------------------+-------------------------------------------------
     Barrett 2012 |         -0.127      -0.476       0.223      7.59
     Barrett 2018 |         -0.014      -0.250       0.221      9.56
     Carmody 2011 |         -0.301      -0.700       0.098      6.81
 Christopher 2018 |         -0.464      -0.861      -0.067      6.84
   Dvorakova 2017 |         -0.361      -0.683      -0.038      8.04
     Greeson 2014 |         -0.529      -0.888      -0.171      7.44
       Hwang 2019 |         -0.369      -0.708      -0.030      7.76
       Klatt 2009 |         -0.198      -0.652       0.255      6.02
       Klatt 2017 |         -0.621      -1.062      -0.180      6.19
         Lee 2010 |         -0.783      -1.194      -0.372      6.62
    Moynihan 2013 |         -0.142      -0.363       0.080      9.81
        Oken 2010 |         -0.450      -1.188       0.287      3.29
        Park 2016 |         -0.647      -1.044      -0.250      6.84
      Wilson 2012 |         -1.022      -1.396      -0.648      7.19
------------------+-------------------------------------------------
            theta |         -0.409      -0.581      -0.236
--------------------------------------------------------------------
95% prediction interval for theta: [-0.936, 0.119]

Test of theta = 0: t(13) = -5.12                 Prob > |t| = 0.0002
Test of homogeneity: Q = chi2(13) = 34.87          Prob > Q = 0.0009
```

Table 19. Comparison of MBPs with passive control groups: univariate meta-analysis of psychosomatic symptoms at 1-6 months follow-up.

```
Meta-analysis summary                     Number of studies =      7
Random-effects model                      Heterogeneity:
Method: REML                                           tau2 =  0.1332
SE adjustment: Truncated Knapp-Hartung               I2 (%) =   79.31
                                                         H2 =    4.83

--------------------------------------------------------------------
            Study |    Effect Size    [95% Conf. Interval]  % Weight
------------------+-------------------------------------------------
     Barrett 2012 |         -0.026      -0.414       0.363     14.53
     Barrett 2018 |         -0.010      -0.246       0.226     16.96
     Carmody 2011 |         -0.233      -0.674       0.207     13.64
 Christopher 2018 |         -0.223      -0.785       0.339     11.63
       Hwang 2019 |         -0.293      -0.693       0.107     14.33
    Moynihan 2013 |          0.098      -0.180       0.375     16.35
      Wilson 2012 |         -1.239      -1.742      -0.735     12.57
------------------+-------------------------------------------------
            theta |         -0.245      -0.645       0.155
--------------------------------------------------------------------
95% prediction interval for theta: [-1.273, 0.783]

Test of theta = 0: t(6) = -1.50                  Prob > |t| = 0.1846
Test of homogeneity: Q = chi2(6) = 23.43           Prob > Q = 0.0007
```

Table 20. Comparison of MBPs with active non-specific control groups: multivariate meta-analysis of psychosomatic symptoms at post-intervention, (Post-int), and 1-6 months follow-up (1-6m). Exchangeable between-study variance-covariance matrices (0.5).

```
Multivariate meta-analysis
Variance-covariance matrix = proportional .5*I(2)+.5*J(2,2,1)
Method = reml                                Number of dimensions    =     2
Restricted log likelihood = -.11828547       Number of observations  =     2
```

	Coef.	Std. Err.	z	P>|z|	[95% Conf. Interval]	
Overall mean						
Post-int	-.358	.195	-1.83	0.067	-.742	.024
1-6m	-.291	.302	-0.96	0.336	-.884	.302

Table 21. Comparison of MBPs with active specific control groups: multivariate meta-analysis of psychosomatic symptoms at post-intervention, (post-int), 1-6 months follow-up (1-6m), and 6+ months follow-up (+6m). Exchangeable between-study variance-covariance matrices (0.8).

```
Multivariate meta-analysis
Variance-covariance matrix = proportional .2*I(3)+.8*J(3,3,1)
Method = reml                                 Number of dimensions    =     3
Restricted log likelihood = -1.3820193        Number of observations  =     7
```

	Coef.	Std. Err.	z	P>|z|	[95% Conf. Interval]	
Overall mean						
Post-int	-.181	.099	-1.82	0.068	-.376	.013
1-6m	-.133	.116	-1.15	0.250	-.360	.093
+6m	-.146	.139	-1.05	0.292	-.420	.126

Table 22. Comparison of MBPs with passive control groups: multivariate meta-analysis of dispositional mindfulness at post-intervention, (post-int), 1-6 months follow-up (1-6m), and 6+ months follow-up (+6m). Exchangeable between-study variance-covariance matrices (0.9).

```
Multivariate meta-analysis
Variance-covariance matrix = proportional .1*I(3)+.9*J(3,3,1)
Method = reml                                Number of dimensions    =     3
Restricted log likelihood = -40.775514       Number of observations  =    52
```

	Coef.	Std. Err.	z	P>|z|	[95% Conf. Interval]	
Overall mean						
Post-int	.538	.068	7.91	0.000	.405	.672
1-6m	.559	.080	6.95	0.000	.401	.716
+6m	.518	.141	3.67	0.000	.241	.795

Table 23. Comparison of MBPs with active non-specific control groups: multivariate meta-analysis of dispositional mindfulness at post-intervention, (post-int), and 1-6 months follow-up (1-6m).

```
Multivariate meta-analysis
Variance-covariance matrix = unstructured
Method = reml                                  Number of dimensions    =     2
Restricted log likelihood = -4.2903167         Number of observations  =     8
```

	Coef.	Std. Err.	z	P>|z|	[95% Conf.	Interval]
Overall mean						
Post-int	.212	.116	1.83	0.068	-.015	.440
1-6m	.274	.171	1.60	0.110	-.062	.611

Table 24. Comparison of MBPs with active specific control groups: multivariate meta-analysis of dispositional mindfulness at post-intervention, (post-int), 1-6 months follow-up (1-6m), and 6+ months follow-up (+6m). Exchangeable between-study variance-covariance matrices (0.9).

```
Multivariate meta-analysis
Variance-covariance matrix = proportional .1*I(3)+.9*J(3,3,1)
Method = reml                                  Number of dimensions    =     3
Restricted log likelihood = -7.8690442         Number of observations  =    19
```

	Coef.	Std. Err.	z	P>|z|	[95% Conf. Interval]	
Overall mean						
Post-int	.135	.080	1.67	0.094	-.023	.293
1-6m	.122	.096	1.27	0.204	-.066	.312
+6m	.153	.123	1.25	0.212	-.087	.395

Table 25. Risk of bias assessment for individual studies.

Study	D1	D2	D3	D4	D5
Aeamla-Or 2015	Low	Low	Low	High	Some
Agee 2009	Some	High	High	High	High
Allen 2012	Some	High	High	Some	Some
Amutio 2015	Some	High	High	High	Some
Anclair 2018	Some	High	Some	High	Some
Anderson 2007	Some	High	High	Some for test, high for self-report	Some
Armstrong 2016	Low	Some	High	High	Some
Arredondo 2017	Some	High	High	High	Some
Astin 1997	Some	High	High	High	Some
Asuero 2014	Some	High	High	High	Some
Auseron 2018	Low	Some	High	High	High
Barrett 2012	Low	High	Low	High	Some
Barrett 2018	Low	Some	Low	High	Low
Beattie 2017	Low	Some	High	High	Low
Behbahani 2018	Some	High	High	High	Some
Benn 2012	Some	High	High	High	Some
Berghmans 2010	Some	High	High	High	Some
Black 2015	Low	Some	Some	High	Some
Brown 2016	Some	Some	Low	High	Some
Carmody 2011	Low	High	High	High	Some
Carson 2004*	Some for D1, Low for D2	high	high	high	some
Cerna 2019	Low	High	High	High	Some
Christopher 2018	Some	High	Some	High	Some
Cohen-Katz 2004	Some	High	High	High	Some
Corsica 2014	Some	High	High	High	Some
Cousin 2016	Some	High	High	High	Some
DamiãoNeto 2019	Some	Some	High	High	Some
Davidson 2003	Some	High	High	High	Some
Delgado 2010	Some	High	High	High	Some
Delgado-Pastor 2015	Some	High	High	High	Some
Desbordes 2012	Some	High	High	High	Some
DeVibe 2013	Low	High	High	High	Some
Duncan 2017	Low	Low	Low	Low for test, high for self-report	Some
Dvorakova 2017	Some	High	Low	High	Some
Dykens 2014	Some	High	Some	High	High
Dziok 2010	Some	High	High	High	Some
Esch 2017	Low	High	Low	Low for test, high for self-report	Some
Ferraioli 2013	Some	High	High	High	Some
Fiocco 2018	Some	Some	High	High	Some
Flook 2013	Some	High	High	High	Some
Frisvold 2009	Some	Some	Low	High	Some
Galante 2018	Low	Some	Low	Low for test, high for self-report	Low

Gallego 2014	Some	High	High	High	Some
Gambrel 2015*	Some for D1, Low for D2	high	high	high	some
Giannandrea 2018	Some	High	High	High	Some
Glass 2019	Some	High	High	High	Some
Grandpierre 2013	Some	High	High	High	Some
Greenberg 2010	Some	High	High	Some	Some
Greeson 2014	Some	High	Some	High	Some
Guardino 2014	Some	Some	High	High	Some
Haarig 2016	Some	High	Low	High	Some
Hou 2013	Low	High	High	High	Some
Huang 2015	Low	High	Low	High	Some
Hunt 2018	Some	High	High	High	Some
Hwang 2019*	Low for D1, Some for D2	some	some	high	some
Ireland 2017	Some	High	Some	High	Some
Isbel 2019	Some	High	High	Some for test, high for self-report	Some
Jain 2007	Some	High	High	High	Some
James 2018	Low	Some	High	High	Some
Josefsson 2014	Some	High	High	Some for test, high for self-report	Some
Kang 2009	Low	High	High	High	Some
Kaviani 2008	Some	High	Low	High	Some
Kingston 2007	Low	Some	High	High	Some
Kirk 2016	Some	High	High	High	Some
Klatt 2009	Some	High	High	High	Some
Klatt 2017	Some	High	Some	High	Some
Kor 2019	Low	Some	Low	High	Low
Krick 2019	Some	Some	Low	High	Some
Kuhlmann 2016	Low	High	High	High	Low
Lacerda 2018	Some	High	High	High	Some
Lara-Cinisomo 2019	Some	High	Low	High	Some
Lebares 2019	Some	High	Low	Low for test, high for self-report	Some
Lee 2010	Some	High	High	High	Some
Li 2018	Some	High	High	High	Some
Lin 2019	Some	High	High	High	Some
Liu 2013	Some	High	High	High	Some
Liu 2015	Some	High	High	High	Some
Lo 2017	Some	High	Low	High	Some
Lonnberg 2020	Low	Some	Low	High	Some
Lopez-Maya 2019	Low	Some	Some	High	Some
Lynch 2018	Some	High	High	High	Some
Ma 2019	Some	Some	Low	Low for test, high for self-report	Some
MacCoon 2012	Low	Some	Some	Low for test, high for self-report	High
Malarkey 2013	Some	Some	High	High	Some
Malinowski 2017	Some	Some	High	Some for test, high for self-report	Some
Manotas 2014	Some	High	High	High	Some

Moody 2013	Some	High	High	High	Some
Moritz 2006	Low	High	High	High	Some
Moynihan 2013*	High for D1, Low for D2	high	some	high	some
Mrazek 2013	Some	High	High	High	Some
Neece 2014*	Low for D1, Low for D2	high	some	high	some
Norouzi 2020	Some	Some	Low	High	Some
Nyklicek 2008	Some	High	High	High	Some
ODonnell 2017	Some	High	Some	High	Some
Oken 2010	Some	High	High	Low for test, high for self-report	Some
Pan 2018	Low	High	Low	High	Some
Park 2016	Some	High	High	High	Some
Perez-Blasco 2013	Some	High	Some	High	Some
Perez-Blasco 2016	Some	High	Low	High	Some
Phang 2015	Low	High	Low	High	Some
Pipe 2009	Some	Some	Low	High	Some
Plummer 2018	Some	High	High	High	Some
Pots 2014	Low	High	Low	High	Some
Prakash 2015	Some	Some	Low	Low for test, high for self-report	Some
Richards 2012	Some	High	High	High	Some
Richards 2013	Some	High	Some	High	Some
Robins 2012	Some	High	High	High	Some
Roeser 2013	Some	High	High	Some for test, high for self-report	Some
Sampl 2017	Some	High	High	Low for test, high for self-report	Some
Schellekens 2017	Low	High	Low	High	Low
Schroeder 2018	Some	High	High	Low for test, high for self-report	Some
Sevinc 2018*	Low for D1, Low for D2	some	low	high	some
Shapiro 1998	Some	High	High	High	Some
Shapiro 2005	Some	High	High	High	Some
Shapiro 2019	Some	High	High	High	Some
Shearer 2016	Some	High	High	High	Some
Smart 2017	Some	High	High	High	Some
Ştefan 2018	Some	High	High	High	Some
Steinberg 2016	Some	High	Low	High	Some
Strub 2013	Some	High	Low	High	Some
Thomas 2016	Some	High	Some	High	Some
VanBerkel 2014	Some	High	Some	Low for test, high for self-report	Some
VanDam 2014	Some	High	High	High	Some
vanDijk 2017*	Low for D1, Low for D2	some	low	high	some
Verweij 2018	Some	High	Low	High	Some
Vieten 2008	Some	High	Low	High	Some
Vinesett 2017	Some	Some	High	High	Some
Wang 2012	Some	High	Low	High	Some
Whitebird 2013	Some	High	Low	High	Some
Williams 2001	Some	High	High	High	Some

Wilson 2012	Some	High	High	High	Some
Wong 2018	Low	Some	Some	High	Some
Woolhouse 2014	Low	High	Some	High	Some
Xu 2015	Some	High	High	High	Some
Yazdanimehr 2016	Some	High	High	High	Some
Zhang 2018	Some	High	High	High	Some

The RoB2 tool measures potential bias across five sources (called 'domains' in the tool): (D1) randomisation, (D2) deviations from intended interventions, (D3) missing outcome data, (D4) measurement of the outcome, and (D5) selection of the reported result. * Cluster RCTs, which were assessed with their specific sub-set of questions [58]. Abbreviations: high: high risk; low: low risk, some: some concerns.

Table 26. Potentially eligible trial registry records with no available results

Title	Identification details	Recruitment target	Control group type	Likelihood of having measured primary outcomes
MBSR Improves Memory and Attention Due to a Stress Reduction as Opposed to Specific Memory Training	NCT02672761	140	Passive & active	Unlikely
Effects of Mindfulness Training on Emotion Regulation and Social Cognition, a Psychophysiological and Neuroimaging Randomized Controlled Study.	NCT03035669	60	Active	Unlikely
The Mindfulness Intervention and Repeated Acute Stress (MIRAS) Study	NCT02894229	150	Passive & active	Unlikely
Promoting Mental Well-being of Pregnant Women with Mindfulness-Based Childbirth and Parenting (MBCP) in Hong Kong	ChiCTR-TRC-13004070	178	Active	Time point not specified
Mindfulness-based Training in the Workplace - evaluating the cost effectiveness and impact on emotional wellbeing	ISRCTN03386834	60	Passive	Likely
The Effects of Well-being Interventions on Affect, Attention, Sleep, Social Stress and Pain Regulation	NCT01057368	161	Passive & active	Time point and outcomes not specified

Table 27. Grading of Recommendations Assessment, Development and Evaluation (GRADE) assessment details by confidence domain: risk of bias, non-reporting bias, imprecision, inconsistency, and indirectness.

Control group	Review outcome	Time point	RoB	Non-rep bias	Imprecision	Inconsistency	Indirectness	GRADE confidence
PC	Anxiety	1-6m	Serious	Not serious	Not serious	Not serious	Not serious	Moderate
PC	Depression	1-6m	Serious	Not serious	Serious	Not serious	Not serious	Low
PC	Distress	1-6m	Serious	Not serious	Not serious	Serious	Not serious	Low
PC	Wellbeing	1-6m	Serious	Serious	Not serious	Serious	Not serious	Very low
NC	Anxiety	1-6m	Serious	Serious	Not serious	Serious	Not serious	Very low
NC	Depression	1-6m	Serious	Not serious	Not serious	Serious	Not serious	Low
NC	Distress	1-6m	Serious	Serious	Serious	Serious	Not serious	Very low
NC	Wellbeing	1-6m	Serious	Not serious	Not serious	Serious	Serious	Very Low
AC	Anxiety	1-6m	Serious	Not serious	Serious	Serious	Not serious	Very low
AC	Depression	1-6m	Serious	Not serious	Not serious	Serious	Not serious	Low
AC	Distress	1-6m	Serious	Serious	Serious	Serious	Not serious	Very low
AC	Wellbeing	1-6m	Serious	Serious	Serious	Serious	Not serious	Very low

Abbreviations: RoB=Risk of bias, 1-6m= 1 to 6 months post-intervention, non-rep: non-reporting, PC=passive controls, NC=active non-specific controls, AC=active specific controls.

Table 28. Sensitivity analysis of methodological quality removing high-risk-of-bias trials. Comparison of MBPs with passive control groups: multivariate meta-analysis of anxiety, depression, distress, and wellbeing at post-intervention, (post-int), 1-6 months follow-up, (1-6m) and 6+ months follow-up (+6m).

```
Multivariate meta-analysis
Variance-covariance matrix = proportional .9*I(11)+.1*J(11,11,1)
Method = reml                              Number of dimensions    =    11
Restricted log likelihood = -52.49954      Number of observations  =    29
```

	Coef.	Std. Err.	z	P>|z|	[95% Conf.	Interval]
Overall mean						
Post-int anxiety	-.589	.114	-5.15	0.000	-.813	-.364
1-6m anxiety	-.218	.179	-1.22	0.224	-.569	.133
Post-int depression	-.267	.086	-3.10	0.002	-.436	-.097
1-6m depression	-.244	.126	-1.93	0.054	-.492	.003
+6m depression	-.144	.272	-0.53	0.596	-.677	.389
Post-int distress	-.381	.069	-5.49	0.000	-.518	-.245
1-6m distress	-.295	.092	-3.19	0.001	-.476	-.114
+6m distress	-.178	.162	-1.10	0.271	-.496	.139
Post-int wellbeing	.354	.102	3.48	0.001	.154	.554
1-6m wellbeing	.269	.143	2.07	0.038	.015	.577
+6m wellbeing	.261	.163	1.60	0.109	-.057	.581

Table 29. Sensitivity analysis of methodological quality removing high-risk-of-bias trials. Comparison of MBPs with active non-specific control groups: multivariate meta-analysis of anxiety, depression, distress, and wellbeing at post-intervention, (post-int), 1-6 months follow-up, (1-6m) and 6+ months follow-up (+6m).

```
Multivariate meta-analysis
Variance-covariance matrix = proportional .3*I(8)+.7*J(8,8,1)
Method = reml                              Number of dimensions    =     8
Restricted log likelihood = -22.551656     Number of observations  =    10
```

	Coef.	Std. Err.	z	P>|z|	[95% Conf. Interval]	
Overall mean						
Post-int anxiety	-.675	.266	-2.54	0.011	-1.197	-.153
1-6m anxiety	-.553	.262	-2.11	0.035	-1.068	-.038
Post-int depression	-.435	.216	-2.01	0.044	-.860	-.010
1-6m depression	-.463	.224	-2.07	0.039	-.904	-.023
Post-int distress	-.398	.199	-2.00	0.046	-.790	-.007
1-6m distress	-.096	.233	-0.41	0.680	-.555	.361
Post-int wellbeing	3.278	.705	4.65	0.000	1.897	4.660
1-6m wellbeing	1.546	.573	2.70	0.007	.422	2.669

Table 30. Sensitivity analysis of methodological quality removing high-risk-of-bias trials. Comparison of MBPs with active specific control groups: multivariate meta-analysis of anxiety, depression, distress, and wellbeing at post-intervention, (post-int), 1-6 months follow-up, (1-6m) and 6+ months follow-up (+6m).

```
Multivariate meta-analysis
Variance-covariance matrix = proportional .9*I(11)+.1*J(11,11,1)
Method = reml                              Number of dimensions    =    11
Restricted log likelihood = -14.333741     Number of observations  =    17
```

	Coef.	Std. Err.	z	P>|z|	[95% Conf.	Interval]
Overall mean						
Post-int anxiety	-.066	.154	-0.43	0.666	-.370	.236
1-6m anxiety	.377	.206	1.83	0.068	-.027	.782
Post-int depression	-.303	.076	-3.95	0.000	-.453	-.153
1-6m depression	-.212	.087	-2.44	0.015	-.382	-.041
+6m depression	-.032	.146	-0.22	0.826	-.319	.255
Post-int distress	-.168	.068	-2.47	0.014	-.302	-.034
1-6m distress	-.048	.085	-0.56	0.573	-.216	.119
+6m distress	.045	.144	0.32	0.751	-.237	.329
Post-int wellbeing	.086	.105	0.82	0.411	-.120	.293
1-6m wellbeing	.032	.128	0.25	0.799	-.219	.285
+6m wellbeing	-.011	.185	-0.06	0.949	-.376	.352

Table 31. Sensitivity analysis of methodological quality removing trials at high risk of bias due to deviations from intended interventions. Comparison of MBPs with passive control groups: multivariate meta-analysis of anxiety, depression, distress, and wellbeing at post-intervention, (post-int), 1-6 months follow-up, (1-6m) and 6+ months follow-up (+6m).

```
Multivariate meta-analysis
Variance-covariance matrix = proportional .9*I(11)+.1*J(11,11,1)
Method = reml                               Number of dimensions    =    11
Restricted log likelihood = -3.302925       Number of observations  =     8
```

	Coef.	Std. Err.	z	P>|z|	[95% Conf.	Interval]
Overall mean						
Post-int anxiety	-.403	.184	-2.19	0.029	-.765	-.042
1-6m anxiety	.089	.134	0.67	0.506	-.174	.352
Post-int depression	-.262	.110	-2.37	0.018	-.479	-.045
1-6m depression	-.310	.112	-2.77	0.006	-.529	-.090
+6m depression	-.222	.161	-1.37	0.170	-.539	.095
Post-int distress	-.407	.068	-5.96	0.000	-.541	-.273
1-6m distress	-.394	.081	-4.81	0.000	-.554	-.233
+6m distress	-.213	.102	-2.09	0.036	-.414	-.013
Post-int wellbeing	.227	.101	2.25	0.025	.029	.426
1-6m wellbeing	.317	.100	3.15	0.002	.119	.515
+6m wellbeing	.258	.104	2.48	0.013	.054	.462

Table 32. Sensitivity analysis of within-study correlation assumptions using Riley's method. Comparison of MBPs with passive control groups: multivariate meta-analysis of anxiety, depression, distress, and wellbeing at post-intervention, (post-int), 1-6 months follow-up, (1-6m) and 6+ months follow-up (+6m).

```
Multivariate meta-analysis
Variance-covariance matrix = proportional .9*I(11)+.1*J(11,11,1)
Method = reml                               Number of dimensions    =    11
Restricted log likelihood = -141.86393      Number of observations  =    78
```

	Coef.	Std. Err.	z	P>|z|	[95% Conf.	Interval]
Overall mean						
Post-int anxiety	-.708	.101	-6.96	0.000	-.907	-.508
1-6m anxiety	-.685	.150	-4.55	0.000	-.981	-.390
Post-int depression	-.465	.074	-6.18	0.000	-.610	-.320
1-6m depression	-.580	.117	-4.96	0.000	-.810	-.351
+6m depression	-.089	.371	-0.24	0.810	-.816	.638
Post-int distress	-.447	.055	-8.10	0.000	-.555	-.338
1-6m distress	-.454	.081	-5.56	0.000	-.614	-.294
+6m distress	-.125	.195	-0.64	0.522	-.509	.258
Post-int wellbeing	.366	.083	4.39	0.000	.203	.530
1-6m wellbeing	.268	.133	2.00	0.045	.005	.530
+6m wellbeing	.200	.217	0.92	0.355	-.225	.626

Table 33. Sensitivity analysis of within-study correlation assumptions using Riley's method. Comparison of MBPs with active non-specific control groups: multivariate meta-analysis of anxiety, depression, distress, and wellbeing at post-intervention, (post-int), 1-6 months follow-up, (1-6m) and 6+ months follow-up (+6m).

```
Multivariate meta-analysis
Variance-covariance matrix = proportional .3*I(8)+.7*J(8,8,1)
Method = reml                                  Number of dimensions    =     8
Restricted log likelihood = -24.812373         Number of observations  =    11
```

	Coef.	Std. Err.	z	P>|z|	[95% Conf.	Interval]
Overall mean						
Post-int anxiety	-.587	.222	-2.64	0.008	-1.023	-.151
1-6m anxiety	-.422	.217	-1.94	0.053	-.849	.004
Post-int depression	-.445	.191	-2.33	0.020	-.820	-.071
1-6m depression	-.436	.192	-2.27	0.023	-.813	-.059
Post-int distress	-.390	.177	-2.20	0.028	-.739	-.042
1-6m distress	-.111	.200	-0.56	0.578	-.504	.281
Post-int wellbeing	4.824	.429	11.24	0.000	3.983	5.666
1-6m wellbeing	2.212	.370	5.98	0.000	1.487	2.938

Table 34. Sensitivity analysis of within-study correlation assumptions using Riley's method. Comparison of MBPs with active specific control groups: multivariate meta-analysis of anxiety, depression, distress, and wellbeing at post-intervention, (post-int), 1-6 months follow-up, (1-6m) and 6+ months follow-up (+6m).

```
Multivariate meta-analysis
Variance-covariance matrix = proportional .9*I(11)+.1*J(11,11,1)
Method = reml                                Number of dimensions    =    11
Restricted log likelihood = -18.786589       Number of observations  =    31
```

	Coef.	Std. Err.	z	P>|z|	[95% Conf.	Interval]
Overall mean						
Post-int anxiety	-.133	.090	-1.48	0.139	-.310	.043
1-6m anxiety	-.020	.181	-0.11	0.909	-.377	.335
Post-int depression	-.289	.060	-4.75	0.000	-.408	-.169
1-6m depression	-.210	.078	-2.70	0.007	-.364	-.057
+6m depression	-.042	.145	-0.29	0.771	-.327	.242
Post-int distress	-.092	.051	-1.79	0.073	-.193	.008
1-6m distress	-.056	.072	-0.78	0.434	-.198	.085
+6m distress	-.093	.126	-0.74	0.462	-.340	.154
Post-int wellbeing	.110	.070	1.57	0.116	-.027	.249
1-6m wellbeing	-.021	.113	-0.19	0.850	-.243	.200
+6m wellbeing	-.028	.165	-0.17	0.864	-.351	.295

Table 35. Sensitivity analysis of within study correlation assumptions. Univariate meta-analyses of anxiety, depression, distress and wellbeing outcomes comparing MBPs with passive control groups.

Anxiety

```
Meta-analysis summary                     Number of studies =      8
Random-effects model                      Heterogeneity:
Method: REML                                           tau2 =  0.5671
SE adjustment: Truncated Knapp-Hartung               I2 (%) =   95.11
                                                         H2 =   20.47

--------------------------------------------------------------------
            Study |    Effect Size    [95% Conf. Interval]  % Weight
------------------+-------------------------------------------------
     Carmody 2011 |         -0.487      -0.789      -0.184     13.35
 Christopher 2018 |          0.190      -0.262       0.642     12.72
       Dziok 2010 |         -1.008      -1.682      -0.335     11.51
     Kaviani 2008 |         -2.427      -3.286      -1.567     10.39
         Kor 2019 |          0.020      -0.092       0.132     13.83
       Sampl 2017 |         -0.827      -1.117      -0.537     13.39
      VanDam 2014 |         -1.153      -1.700      -0.607     12.23
 Yazdanimehr 2016 |         -1.474      -1.952      -0.997     12.59
------------------+-------------------------------------------------
            theta |         -0.844      -1.525      -0.162
--------------------------------------------------------------------
95% prediction interval for theta: [-2.817, 1.129]

Test of theta = 0: t(7) = -2.93                Prob > |t| = 0.0221
Test of homogeneity: Q = chi2(7) = 108.83        Prob > Q = 0.0000
```

Depression

```
Meta-analysis summary                     Number of studies =     14
Random-effects model                      Heterogeneity:
Method: REML                                           tau2 =  0.6351
SE adjustment: Truncated Knapp-Hartung               I2 (%) =   93.22
                                                         H2 =   14.76

--------------------------------------------------------------------
            Study |    Effect Size    [95% Conf. Interval]  % Weight
------------------+-------------------------------------------------
   Aeamla-Or 2015 |         -0.334      -0.639      -0.028      7.69
     Barrett 2018 |         -0.015      -0.251       0.221      7.80
        Benn 2012 |         -0.302      -0.814       0.210      7.21
     Carmody 2011 |         -0.296      -0.672       0.080      7.54
 Christopher 2018 |          0.296      -0.167       0.760      7.34
       Dziok 2010 |         -0.949      -1.890      -0.008      5.86
      Haarig 2016 |         -0.843      -1.473      -0.212      6.86
     Kaviani 2008 |         -1.984      -2.794      -1.174      6.29
         Kor 2019 |         -0.571      -1.040      -0.102      7.32
    Moynihan 2013 |          0.000      -0.277       0.277      7.74
      Roeser 2013 |         -1.118      -1.530      -0.707      7.46
      VanDam 2014 |         -1.258      -1.679      -0.838      7.44
      Vieten 2008 |          0.566      -0.163       1.295      6.55
 Yazdanimehr 2016 |         -2.604      -3.226      -1.982      6.89
------------------+-------------------------------------------------
            theta |         -0.649      -1.143      -0.155
--------------------------------------------------------------------
95% prediction interval for theta: [-2.455, 1.158]

Test of theta = 0: t(13) = -2.84               Prob > |t| = 0.0140
Test of homogeneity: Q = chi2(13) = 128.63       Prob > Q = 0.0000
```

Distress

```
Meta-analysis summary                     Number of studies =     27
Random-effects model                      Heterogeneity:
Method: REML                                           tau2 =  0.0745
SE adjustment: Truncated Knapp-Hartung               I2 (%) =   66.97
                                                         H2 =    3.03
```

Study	Effect Size	[95% Conf.	Interval]	% Weight
Aeamla-Or 2015	-0.337	-0.660	-0.015	4.55
Arredondo 2017	-1.474	-2.254	-0.693	1.98
Barrett 2012	-0.106	-0.476	0.265	4.19
Barrett 2018	-0.021	-0.257	0.215	5.20
Behbahani 2018	-0.713	-1.233	-0.192	3.19
Benn 2012	-0.672	-1.241	-0.102	2.91
Carmody 2011	-0.512	-0.848	-0.175	4.45
Carson 2004	-0.741	-1.147	-0.336	3.94
Christopher 2018	-0.085	-0.597	0.427	3.24
Davidson 2003	0.000	-0.627	0.627	2.61
Galante 2018	-0.419	-0.581	-0.257	5.68
Huang 2015	-0.449	-0.755	-0.143	4.67
Hwang 2019	-0.454	-0.830	-0.078	4.15
Kor 2019	-0.838	-1.438	-0.238	2.75
Lin 2019	-0.557	-0.950	-0.165	4.04
Moritz 2006	-0.385	-0.738	-0.033	4.33
Moynihan 2013	0.000	-0.277	0.277	4.89
Phang 2015	-0.247	-0.674	0.180	3.79
Plummer 2018	-0.397	-0.756	-0.038	4.28
Sampl 2017	-0.860	-1.213	-0.507	4.32
Schellekens 2017	0.197	-0.451	0.844	2.52
Schroeder 2018	-0.922	-1.648	-0.197	2.19
VanDam 2014	-0.613	-1.291	0.065	2.38
Vieten 2008	0.580	-0.150	1.309	2.17
Wilson 2012	-1.022	-1.468	-0.577	3.67
Yazdanimehr 2016	-1.040	-1.502	-0.578	3.55
vanDijk 2017	-0.412	-0.760	-0.064	4.36
theta	-0.446	-0.597	-0.295	

```
95% prediction interval for theta: [-1.028, 0.136]

Test of theta = 0: t(26) = -6.08                Prob > |t| = 0.0000
Test of homogeneity: Q = chi2(26) = 73.49         Prob > Q = 0.0000
```

Wellbeing

```
Meta-analysis summary                     Number of studies =      9
Random-effects model                      Heterogeneity:
Method: REML                                          tau2 =  0.0267
SE adjustment: Truncated Knapp-Hartung              I2 (%) =   53.18
                                                        H2 =    2.14

--------------------------------------------------------------------
            Study |    Effect Size    [95% Conf. Interval]  % Weight
------------------+-------------------------------------------------
     Barrett 2012 |          0.190      -0.163       0.543     10.59
     Barrett 2018 |          0.019      -0.217       0.254     15.20
        Benn 2012 |          0.319      -0.228       0.865      5.99
    Davidson 2003 |          0.000      -0.627       0.627      4.84
     Galante 2018 |          0.286       0.130       0.443     18.94
         Lin 2019 |          0.655       0.267       1.042      9.52
    Moynihan 2013 |          0.079      -0.167       0.325     14.75
      Wilson 2012 |          0.592       0.171       1.014      8.57
     vanDijk 2017 |          0.512       0.189       0.835     11.60
------------------+-------------------------------------------------
            theta |          0.280       0.098       0.463
--------------------------------------------------------------------
95% prediction interval for theta: [-0.149, 0.710]

Test of theta = 0: t(8) = 3.55                    Prob > |t| = 0.0076
Test of homogeneity: Q = chi2(8) = 15.79            Prob > Q = 0.0456
```

Table 36. Sensitivity analysis of within study correlation assumptions. Univariate meta-analyses of anxiety, depression, distress and wellbeing outcomes compared with active non-specific control groups.

Anxiety

```
Meta-analysis summary                     Number of studies =      4
Random-effects model                      Heterogeneity:
Method: REML                                          tau2 =  0.6123
SE adjustment: Truncated Knapp-Hartung              I2 (%) =   93.16
                                                        H2 =   14.61

--------------------------------------------------------------------
            Study |    Effect Size    [95% Conf. Interval]  % Weight
------------------+-------------------------------------------------
   Armstrong 2016 |         -0.336      -0.889       0.217     23.99
         Hou 2013 |         -0.257      -0.575       0.060     25.99
       James 2018 |         -0.156      -0.509       0.197     25.74
     Norouzi 2020 |         -1.900      -2.424      -1.377     24.28
------------------+-------------------------------------------------
            theta |         -0.649      -1.956       0.658
--------------------------------------------------------------------
95% prediction interval for theta: [-4.452, 3.154]

Test of theta = 0: t(3) = -1.58                  Prob > |t| = 0.2122
Test of homogeneity: Q = chi2(3) = 33.69           Prob > Q = 0.0000
```

Depression

```
Meta-analysis summary                     Number of studies =      6
Random-effects model                      Heterogeneity:
Method: REML                                          tau2 =  0.3364
SE adjustment: Truncated Knapp-Hartung              I2 (%) =   90.31
                                                        H2 =   10.32

--------------------------------------------------------------------
            Study |    Effect Size    [95% Conf. Interval]  % Weight
------------------+-------------------------------------------------
   Armstrong 2016 |         -0.011      -0.538       0.516     15.72
      Duncan 2017 |         -0.804      -1.434      -0.174     14.61
    Frisvold 2009 |         -0.508      -0.702      -0.314     18.56
         Hou 2013 |         -0.418      -0.764      -0.073     17.49
       James 2018 |         -0.125      -0.538       0.287     16.88
     Norouzi 2020 |         -1.738      -2.164      -1.311     16.74
------------------+-------------------------------------------------
            theta |         -0.599      -1.252       0.054
--------------------------------------------------------------------
95% prediction interval for theta: [-2.357, 1.159]

Test of theta = 0: t(5) = -2.36                  Prob > |t| = 0.0650
Test of homogeneity: Q = chi2(5) = 39.23           Prob > Q = 0.0000
```

Distress

```
Meta-analysis summary                     Number of studies =      6
Random-effects model                      Heterogeneity:
Method: REML                                           tau2 =  0.0000
SE adjustment: Truncated Knapp-Hartung               I2 (%) =    0.00
                                                         H2 =    1.00

--------------------------------------------------------------------
            Study |    Effect Size    [95% Conf. Interval]  % Weight
------------------+-------------------------------------------------
       Brown 2016 |         -0.058      -0.670       0.553     10.75
    Frisvold 2009 |          0.003      -0.579       0.584     11.88
    Guardino 2014 |         -0.306      -0.958       0.346      9.44
         Hou 2013 |         -0.241      -0.567       0.084     37.92
       James 2018 |         -0.322      -0.795       0.152     17.94
     Norouzi 2020 |         -0.724      -1.301      -0.148     12.07
------------------+-------------------------------------------------
            theta |         -0.271      -0.534      -0.009
--------------------------------------------------------------------
95% prediction interval for theta: [-0.555, 0.012]

Test of theta = 0: t(5) = -2.66                  Prob > |t| = 0.0452
Test of homogeneity: Q = chi2(5) = 3.78            Prob > Q = 0.5820
```

Wellbeing: only one study available.

Table 37. Sensitivity analysis of within study correlation assumptions. Univariate meta-analyses of anxiety, depression, distress and wellbeing outcomes comparing MBPs with active specific control groups.

Anxiety

```
Meta-analysis summary                     Number of studies =      2
Random-effects model                      Heterogeneity:
Method: REML                                          tau2 =  0.0000
SE adjustment: Truncated Knapp-Hartung              I2 (%) =    0.00
                                                        H2 =    1.00

--------------------------------------------------------------------
            Study |    Effect Size    [95% Conf. Interval]  % Weight
------------------+-------------------------------------------------
      Dykens 2014 |         -0.185      -0.598       0.227     60.92
          Ma 2019 |          0.127      -0.388       0.642     39.08
------------------+-------------------------------------------------
            theta |         -0.063      -2.152       2.026
--------------------------------------------------------------------
95% prediction interval for theta: [     .,     .]

Test of theta = 0: t(1) = -0.39                Prob > |t| = 0.7660
Test of homogeneity: Q = chi2(1) = 0.86          Prob > Q = 0.3539
```

Depression

```
Meta-analysis summary                     Number of studies =      9
Random-effects model                      Heterogeneity:
Method: REML                                          tau2 =  0.0336
SE adjustment: Truncated Knapp-Hartung              I2 (%) =   61.62
                                                        H2 =    2.61

--------------------------------------------------------------------
            Study |    Effect Size    [95% Conf. Interval]  % Weight
------------------+-------------------------------------------------
     Barrett 2018 |          0.002      -0.012       0.016     25.18
     Beattie 2017 |         -0.025      -0.853       0.803      4.00
      Dykens 2014 |         -0.035      -0.446       0.377     10.90
    Lonnberg 2020 |         -0.179      -0.438       0.080     16.59
          Ma 2019 |         -0.774      -1.319      -0.228      7.64
    ODonnell 2017 |         -0.901      -1.751      -0.051      3.83
         Pan 2018 |         -0.396      -0.684      -0.108     15.35
    Vinesett 2017 |          0.265      -0.930       1.460      2.09
   Whitebird 2013 |         -0.296      -0.607       0.016     14.41
------------------+-------------------------------------------------
            theta |         -0.225      -0.441      -0.010
--------------------------------------------------------------------
95% prediction interval for theta: [-0.712, 0.261]

Test of theta = 0: t(8) = -2.42                Prob > |t| = 0.0421
Test of homogeneity: Q = chi2(8) = 24.88         Prob > Q = 0.0016
```

Distress

```
Meta-analysis summary                     Number of studies =     11
Random-effects model                      Heterogeneity:
Method: REML                                          tau2 =  0.0092
SE adjustment: Truncated Knapp-Hartung              I2 (%) =   90.55
                                                        H2 =   10.58

--------------------------------------------------------------------
           Study |    Effect Size    [95% Conf. Interval]  % Weight
-----------------+--------------------------------------------------
    Barrett 2012 |          0.111       0.074       0.149     23.76
    Barrett 2018 |         -0.000      -0.014       0.014     24.57
    Beattie 2017 |          0.147      -0.787       1.081      0.96
    Corsica 2014 |          0.000      -0.709       0.709      1.62
     Dykens 2014 |         -0.013      -0.425       0.399      4.26
   Lonnberg 2020 |         -0.064      -0.359       0.231      7.14
         Ma 2019 |         -0.039      -0.590       0.512      2.58
     Moritz 2006 |          0.117       0.083       0.150     23.97
   Vinesett 2017 |          0.073      -1.114       1.261      0.61
  Whitebird 2013 |         -0.445      -0.875      -0.014      3.96
       Wong 2018 |         -0.269      -0.582       0.044      6.56
-----------------+--------------------------------------------------
           theta |          0.015      -0.092       0.121
--------------------------------------------------------------------
95% prediction interval for theta: [-0.228, 0.257]

Test of theta = 0: t(10) = 0.31                 Prob > |t| = 0.7637
Test of homogeneity: Q = chi2(10) = 70.10         Prob > Q = 0.0000
```

Wellbeing

```
Meta-analysis summary                     Number of studies =      4
Random-effects model                      Heterogeneity:
Method: REML                                          tau2 =  0.0005
SE adjustment: Truncated Knapp-Hartung              I2 (%) =   47.36
                                                        H2 =    1.90

--------------------------------------------------------------------
           Study |    Effect Size    [95% Conf. Interval]  % Weight
-----------------+--------------------------------------------------
    Barrett 2012 |          0.045       0.008       0.081     39.59
    Barrett 2018 |          0.005      -0.009       0.020     59.52
     Dykens 2014 |         -0.007      -0.419       0.405      0.79
   Vinesett 2017 |         -0.137      -1.326       1.052      0.10
-----------------+--------------------------------------------------
           theta |          0.021      -0.039       0.081
--------------------------------------------------------------------
95% prediction interval for theta: [-0.108, 0.149]

Test of theta = 0: t(3) = 1.10                  Prob > |t| = 0.3519
Test of homogeneity: Q = chi2(3) = 3.87           Prob > Q = 0.2756
```

Table 38. Sensitivity analysis excluding data from samples N<30. Comparison of MBPs with passive control groups: multivariate meta-analysis of anxiety, depression, distress, and wellbeing at post-intervention, (post-int), 1-6 months follow-up, (1-6m) and 6+ months follow-up (+6m). The prediction intervals are shown in the second table.

```
Multivariate meta-analysis
Variance-covariance matrix = proportional .9*I(11)+.1*J(11,11,1)
Method = reml                               Number of dimensions    =    11
Restricted log likelihood = -23.015115      Number of observations  =    30
```

	Coef.	Std. Err.	z	P>|z|	[95% Conf.	Interval]
Overall mean						
Post-int anxiety	-.484	.085	-5.86	0.000	-.652	-.317
1-6m anxiety	-.503	.114	-4.39	0.000	-.728	-.278
Post-int depression	-.431	.068	-6.32	0.000	-.564	-.297
1-6m depression	-.436	.094	-4.60	0.000	-.622	-.250
+6m depression	-.247	.178	-1.39	0.166	-.597	.102
Post-int distress	-.442	.043	-10.21	0.000	-.527	-.357
1-6m distress	-.399	.055	-7.20	0.000	-.508	-.290
+6m distress	-.231	.110	-2.10	0.036	-.447	-.015
Post-int wellbeing	.321	.058	5.51	0.000	.207	.436
1-6m wellbeing	.353	.081	4.35	0.000	.194	.513
+6m wellbeing	.280	.110	2.53	0.011	.063	.498

Outcome	Estimate	95% Confidence Int.		95% Prediction Int.	
Post-int anxiety	-.484	-.652	-.317	-.846	-.123
1-6m anxiety	-.503	-.728	-.278	-.897	-.109
Post-int depression	-.431	-.564	-.297	-.776	-.085
1-6m depression	-.436	-.622	-.250	-.806	-.065
+6m depression	-.247	-.597	.102	-.730	.235
Post-int distress	-.442	-.527	-.357	-.770	-.114
1-6m distress	-.399	-.508	-.290	-.735	-.063
+6m distress	-.231	-.447	-.015	-.619	.156
Post-int wellbeing	.321	.207	.436	-.016	.659
1-6m wellbeing	.353	.194	.513	-.003	.710
+6 wellbeing	.280	.063	.498	-.108	.670

Table 39. Sensitivity analysis setting estimate to +1 SE. Comparison of MBPs with passive control groups: multivariate meta-analysis of anxiety, depression, distress, and wellbeing at post-intervention, (post-int), 1-6 months follow-up, (1-6m) and 6+ months follow-up (+6m).

```
Multivariate meta-analysis
Variance-covariance matrix = proportional .9*I(11)+.1*J(11,11,1)
Method = reml                                Number of dimensions    =    11
Restricted log likelihood = -127.22526       Number of observations  =    78
```

	Coef.	Std. Err.	z	P>|z|	[95% Conf.	Interval]
Overall mean						
Post-int anxiety	-.700	.085	-8.21	0.000	-.867	-.533
1-6m anxiety	-.569	.126	-4.49	0.000	-.817	-.321
Post-int depression	-.437	.064	-6.81	0.000	-.563	-.311
1-6m depression	-.525	.101	-5.20	0.000	-.723	-.327
+6m depression	-.177	.319	-0.56	0.578	-.803	.448
Post-int distress	-.431	.048	-8.88	0.000	-.526	-.336
1-6m distress	-.430	.071	-6.01	0.000	-.571	-.290
+6m distress	-.153	.170	-0.90	0.368	-.488	.181
Post-int wellbeing	.356	.073	4.87	0.000	.212	.499
1-6m wellbeing	.349	.116	3.00	0.000	.121	.578
+6m wellbeing	.252	.189	1.33	0.183	-.118	.623

Table 40. Sensitivity analysis setting estimate to +1 SE. Comparison of MBPs with active non-specific control groups: multivariate meta-analysis of anxiety, depression, distress, and wellbeing at post-intervention (post-int), 1-6 months follow-up, (1-6m) and 6+ months follow-up (+6m).

```
Multivariate meta-analysis
Variance-covariance matrix = proportional .3*I(8)+.7*J(8,8,1)
Method = reml                              Number of dimensions    =     8
Restricted log likelihood = -23.852515      Number of observations  =    11
```

	Coef.	Std. Err.	z	P>|z|	[95% Conf. Interval]	
Overall mean						
Post-int anxiety	-.542	.208	-2.61	0.009	-.950	-.134
1-6m anxiety	-.462	.205	-2.25	0.024	-.865	-.060
Post-int depression	-.409	.179	-2.28	0.022	-.761	-.058
1-6m depression	-.443	.183	-2.41	0.016	-.803	-.083
Post-int distress	-.362	.167	-2.17	0.030	-.690	-.035
1-6m distress	-.124	.191	-0.65	0.514	-.500	.250
Post-int wellbeing	3.04	.666	4.57	0.000	1.737	4.350
1-6m wellbeing	1.43	.541	2.64	0.008	.370	2.491

Table 41. Sensitivity analysis setting estimate to +1 SE. Comparison of MBPs with active specific control groups: multivariate meta-analysis of anxiety, depression, distress, and wellbeing at post-intervention, (post-int), 1-6 months follow-up, (1-6m) and 6+ months follow-up (+6m).

```
Multivariate meta-analysis
Variance-covariance matrix = proportional .9*I(11)+.1*J(11,11,1)
Method = reml                              Number of dimensions    =    11
Restricted log likelihood = -16.032582     Number of observations  =    31
```

	Coef.	Std. Err.	z	P>|z|	[95% Conf. Interval]	
Overall mean						
Post-int anxiety	-.099	.078	-1.27	0.204	-.252	.054
1-6m anxiety	.078	.138	0.57	0.568	-.191	.349
Post-int depression	-.235	.058	-4.02	0.000	-.350	-.120
1-6m depression	-.161	.077	-2.08	0.037	-.313	-.009
+6m depression	.043	.141	0.31	0.759	-.233	.320
Post-int distress	-.066	.050	-1.32	0.187	-.165	.032
1-6m distress	.003	.071	0.04	0.965	-.137	.143
+6m distress	-.052	.125	-0.42	0.677	-.297	.192
Post-int wellbeing	.168	.068	2.46	0.014	.033	.302
1-6m wellbeing	.028	.106	0.27	0.788	-.179	.236
+6m wellbeing	.001	.180	0.01	0.991	-.351	.355

Table 42. Sensitivity analysis setting estimate to -1 SE. Comparison of MBPs with passive control groups: multivariate meta-analysis of anxiety, depression, distress, and wellbeing at post-intervention, (post-int), 1-6 months follow-up, (1-6m) and 6+ months follow-up (+6m). The prediction intervals are shown in the second table.

```
Multivariate meta-analysis
Variance-covariance matrix = proportional .9*I(11)+.1*J(11,11,1)
Method = reml                               Number of dimensions    =    11
Restricted log likelihood = -117.17977      Number of observations  =    78
```

	Coef.	Std. Err.	z	P>|z|	[95% Conf. Interval]	
Overall mean						
Post-int anxiety	-.691	.080	-8.62	0.000	-.849	-.534
1-6m anxiety	-.567	.118	-4.78	0.000	-.799	-.334
Post-int depression	-.464	.060	-7.64	0.000	-.584	-.345
1-6m depression	-.545	.095	-5.72	0.000	-.732	-.358
+6m depression	-.199	.295	-0.67	0.500	-.779	.380
Post-int distress	-.461	.045	-10.07	0.000	-.551	-.371
1-6m distress	-.465	.067	-6.88	0.000	-.597	-.332
+6m distress	-.169	.159	-1.06	0.287	-.482	.142
Post-int wellbeing	.332	.068	4.85	0.000	.197	.466
1-6m wellbeing	.298	.108	2.74	0.006	.085	.512
+6m wellbeing	.243	.176	1.38	0.167	-.101	.588

Outcome	Estimate	95% Confidence Int.		95% Prediction Int.	
Post-int anxiety	-.691	-.849	-.534	-1.278	-.104
1-6m anxiety	-.567	-.799	-.334	-1.179	.045
Post-int depression	-.464	-.584	-.345	-1.042	.112
1-6m depression	-.545	-.732	-.358	-1.141	.050
+6m depression	-.199	-.779	.380	-1.015	.616
Post-int distress	-.461	-.551	-.371	-1.033	.110
1-6m distress	-.465	-.597	-.332	-1.045	.115
+6m distress	-.169	-.482	.142	-.818	.478
Post-int wellbeing	.332	.197	.466	-.249	.913
1-6m wellbeing	.298	.085	.512	-.306	.903
+6m wellbeing	.243	-.101	.588	-.421	.908

Table 43. Sensitivity analysis setting estimate to -1 SE. Comparison of MBPs with active non-specific control groups: multivariate meta-analysis of anxiety, depression, distress, and wellbeing at post-intervention, (post-int), 1-6 months follow-up, (1-6m) and 6+ months follow-up (+6m). The prediction intervals are shown in the second table.

```
Multivariate meta-analysis
Variance-covariance matrix = proportional .3*I(8)+.7*J(8,8,1)
Method = reml                              Number of dimensions    =     8
Restricted log likelihood = -23.23405      Number of observations  =    11
```

	Coef.	Std. Err.	z	P>|z|	[95% Conf.	Interval]
Overall mean						
Post-int anxiety	-.564	.202	-2.79	0.005	-.960	-.167
1-6m anxiety	-.486	.199	-2.44	0.015	-.876	-.095
Post-int depression	-.443	.174	-2.55	0.011	-.785	-.102
1-6m depression	-.466	.178	-2.62	0.009	-.815	-.117
Post-int distress	-.394	.162	-2.43	0.015	-.712	-.076
1-6m distress	-.150	.186	-0.81	0.419	-.515	.214
Post-int wellbeing	2.978	.660	4.51	0.000	1.682	4.273
1-6m wellbeing	1.382	.534	2.58	0.010	.334	2.429

Outcome	Estimate	95% Confidence Int.		95% Prediction Int.	
Post-int anxiety	-.564	-.960	-.167	-1.679	.551
1-6m anxiety	-.486	-.876	-.095	-1.598	.626
Post-int depression	-.443	-.785	-.102	-1.534	.646
1-6m depression	-.466	-.815	-.117	-1.560	.627
Post-int distress	-.394	-.712	-.076	-1.475	.686
1-6m distress	-.150	-.515	.214	-1.251	.950
Post-int wellbeing	2.978	1.682	4.273	1.169	4.786
1-6m wellbeing	1.382	.334	2.429	-.198	2.962

Table 44. Sensitivity analysis setting estimate to -1 SE. Comparison of MBPs with active specific control groups: multivariate meta-analysis of anxiety, depression, distress, and wellbeing at post-intervention, (post-int), 1-6 months follow-up, (1-6m) and 6+ months follow-up (+6m).

```
Multivariate meta-analysis
Variance-covariance matrix = proportional .9*I(11)+.1*J(11,11,1)
Method = reml                                Number of dimensions    =    11
Restricted log likelihood = -18.460193       Number of observations  =    31
```

	Coef.	Std. Err.	z	P>|z|	[95% Conf.	Interval]
Overall mean						
Post-int anxiety	-.135	.084	-1.61	0.107	-.300	.029
1-6m anxiety	.064	.148	0.44	0.663	-.227	.356
Post-int depression	-.250	.062	-4.02	0.000	-.372	-.128
1-6m depression	-.172	.082	-2.10	0.036	-.334	-.011
+6m depression	-.137	.149	-0.92	0.360	-.431	.156
Post-int distress	-.084	.053	-1.58	0.115	-.189	.020
1-6m distress	-.023	.076	-0.31	0.759	-.172	.125
+6m distress	-.001	.132	-0.01	0.991	-.260	.257
Post-int wellbeing	.168	.073	2.30	0.021	.025	.312
1-6m wellbeing	.028	.113	0.25	0.805	-.194	.250
+6m wellbeing	-.007	.194	-0.04	0.970	-.387	.372

Table 45 Sensitivity analysis excluding studies with unclear teacher competence. Comparison of MBPs with passive control groups: multivariate meta-analysis of anxiety, depression, distress, and wellbeing at post-intervention, (post-int), 1-6 months follow-up, (1-6m) and 6+ months follow-up (+6m). The prediction intervals are shown in the second table.

```
Multivariate meta-analysis
Variance-covariance matrix = proportional .9*I(11)+.1*J(11,11,1)
Method = reml                                Number of dimensions    =    11
Restricted log likelihood = -85.029503       Number of observations  =    55
```

	Coef.	Std. Err.	z	P>|z|	[95% Conf. Interval]	
Overall mean						
Post-int anxiety	-.646	.110	-5.86	0.000	-.863	-.430
1-6m anxiety	-.814	.182	-4.46	0.000	-1.172	-.456
Post-int depression	-.472	.076	-6.15	0.000	-.622	-.321
1-6m depression	-.485	.120	-4.02	0.000	-.722	-.249
+6m depression	-.177	.300	-0.59	0.555	-.766	.411
Post-int distress	-.415	.055	-7.54	0.000	-.523	-.307
1-6m distress	-.383	.080	-4.74	0.000	-.541	-.224
+6m distress	-.151	.161	-0.93	0.351	-.468	.166
Post-int wellbeing	.371	.074	4.96	0.000	.224	.517
1-6m wellbeing	.322	.110	2.92	0.004	.105	.539
+6m wellbeing	.254	.178	1.43	0.154	-.095	.604

Outcome	Estimate	95% Confidence Int.		95% Prediction Int.	
Post-int anxiety	-.646	-.863	-.430	-1.266	-.027
1-6m anxiety	-.814	-1.172	-.456	-1.498	-.129
Post-int depression	-.472	-.622	-.321	-1.070	.126
1-6m depression	-.485	-.722	-.249	-1.112	.141
+6m depression	-.177	-.766	.411	-1.013	.657
Post-int distress	-.415	-.523	-.307	-1.004	.173
1-6m distress	-.383	-.541	-.224	-.983	.217
+6m distress	-.151	-.468	.166	-.814	.512
Post-int wellbeing	.371	.224	.517	-.226	.968
1-6m wellbeing	.322	.105	.539	-.296	.942
+6m wellbeing	.254	-.095	.604	-.425	.934

Table 46. Sensitivity analysis excluding studies with unclear teacher competence. Comparison of MBPs with active non-specific control groups: multivariate meta-analysis of anxiety, depression, distress, and wellbeing at post-intervention, (post-int), 1-6 months follow-up, (1-6m) and 6+ months follow-up (+6m).

```
Multivariate meta-analysis
Variance-covariance matrix = proportional .3*I(8)+.7*J(8,8,1)
Method = reml                              Number of dimensions    =     8
Restricted log likelihood = -23.080973     Number of observations  =    10
```

	Coef.	Std. Err.	z	P>|z|	[95% Conf. Interval]	
Overall mean						
Post-int anxiety	-.542	.211	-2.56	0.011	-.957	-.126
1-6m anxiety	-.462	.207	-2.22	0.026	-.869	-.054
Post-int depression	-.414	.183	-2.26	0.024	-.773	-.054
1-6m depression	-.443	.186	-2.38	0.017	-.808	-.077
Post-int distress	-.357	.176	-2.03	0.042	-.703	-.012
1-6m distress	-.123	.194	-0.64	0.525	-.505	.257
Post-int wellbeing	3.046	.671	4.54	0.000	1.731	4.362
1-6m wellbeing	1.432	.543	2.64	0.008	.367	2.497

Outcome	Estimate	95% Confidence Int.		95% Prediction Int.	
Post-int axiety	-.542	-.957	-.126	-1.725	.641
1-6m anxiety	-.462	-.869	-.054	-1.641	.717
Post-int depression	-.414	-.773	-.054	-1.571	.743
1-6m depression	-.443	-.808	-.077	-1.603	.717
Post-int distress	-.357	-.703	-.012	-1.509	.793
1-6m distress	-.123	-.505	.257	-1.291	1.043
Post-int wellbeing	3.046	1.731	4.362	1.161	4.932
1-6m wellbeing	1.432	.367	2.497	-.220	3.085

Table 47. Sensitivity analysis excluding studies with unclear teacher competence. Comparison of MBPs with active specific control groups: multivariate meta-analysis of anxiety, depression, distress, and wellbeing at post-intervention, (post-int), 1-6 months follow-up, (1-6m) and 6+ months follow-up (+6m).

```
Multivariate meta-analysis
Variance-covariance matrix = proportional .9*I(11)+.1*J(11,11,1)
Method = reml                                Number of dimensions    =    11
Restricted log likelihood = -11.083879       Number of observations  =    22
```

	Coef.	Std. Err.	z	P>|z|	[95% Conf.	Interval]
Overall mean						
Post-int anxiety	-.058	.101	-0.58	0.562	-.257	.139
1-6m anxiety	.388	.195	1.99	0.047	.005	.770
Post-int depression	-.267	.066	-4.01	0.000	-.398	-.136
1-6m depression	-.207	.084	-2.45	0.014	-.373	-.041
+6m depression	-.055	.138	-0.40	0.687	-.326	.215
Post-int distress	-.129	.057	-2.24	0.025	-.241	-.016
1-6m distress	-.038	.081	-0.47	0.638	-.197	.121
+6m distress	-.034	.122	-0.28	0.776	-.275	.205
Post-int wellbeing	.159	.081	1.94	0.052	-.001	.319
1-6m wellbeing	.004	.138	0.03	0.975	-.267	.276
+6m wellbeing	-.009	.175	-0.05	0.959	-.353	.335

Table 48. Multivariate Meta-regression. Comparison of MBPs with passive control groups, outcomes at 1-6 months follow-up. USA studies= studies from the USA; selective MBP=selective intervention; indicated MBP=indicated intervention; Psychoeducation= adding psychoeducation component and/or non-meditative psychological exercises; Physical exercise= adding physical exercise component; Arts= adding arts component.

Multivariate meta-analysis
Variance-covariance matrix = proportional .9*I(4)+.1*J(4,4,1)
Method = reml — Number of dimensions = 4
Restricted log likelihood = -38.844293 — Number of observations = 31

	Coef.	Std. Err.	z	P>|z|	[95% Conf.	Interval]
1-6m anxiety						
Selective MBP	-.601	.360	-1.67	0.095	-1.307	.103
Indicated MBP	-1.123	.418	-2.68	0.007	-1.943	-.303
USA studies	1.099	.500	2.20	0.028	.119	2.080
Contact hours	.020	.031	0.65	0.514	-.041	.083
Physical exercise	-.716	.476	-1.50	0.133	-1.651	.218
_cons	-.910	.525	-1.73	0.083	-1.940	.118
1-6m depression						
Selective MBP	-1.070	.338	-3.16	0.002	-1.734	-.406
Indicated MBP	-.841	.343	-2.45	0.014	-1.515	-.167
USA studies	1.084	.282	3.84	0.000	.530	1.637
Contact hours	.045	.018	2.48	0.013	.009	.081
Psychoeducation	-.550	.383	-1.44	0.151	-1.302	.201
Physical exercise	-.961	.403	-2.38	0.017	-1.752	-.169
_cons	-1.027	.415	-2.48	0.013	-1.841	-.214
1-6m distress						
Selective MBP	-.063	.167	-0.38	0.703	-.392	.264
Indicated MBP	.099	.299	0.33	0.740	-.487	.685
USA studies	.244	.230	1.06	0.291	-.208	.696
Contact hours	.001	.016	0.09	0.928	-.031	.034
Psychoeducation	-.368	.248	-1.49	0.137	-.855	.117
Physical exercise	.115	.224	0.51	0.607	-.323	.554
Arts	-.744	.525	-1.42	0.157	-1.774	.286
_cons	-.534	.313	-1.71	0.088	-1.148	.0795
1-6m wellbeing						
Selective MBP	.156	.371	0.42	0.673	-.571	.883
USA studies	-.397	.813	-0.49	0.626	-1.991	1.197
Contact hours	-.006	.079	-0.08	0.939	-.1621	.149
Psychoeducation	.355	1.307	0.27	0.786	-2.207	2.918
Physical exercise	.247	.553	0.45	0.655	-.837	1.331
Arts	.689	1.156	0.60	0.551	-1.577	2.956
_cons	.348	.937	0.37	0.710	-1.488	2.185

Table 49. Meta-regression. Comparison of MBPs with active specific control groups, outcome distress at 1-6 months follow-up. Selective MBP=selective intervention; indicated MBP=indicated intervention; Psychoeducation= adding psychoeducation component and/or non-meditative psychological exercises; Physical exercise= adding physical exercise component; Other meditation= adding other types meditation component; Arts= adding arts component. Psychoeducation and other meditation are omitted because of collinearity.

```
Random-effects meta-regression                    Number of obs  =        11
Method: REML                                      Residual heterogeneity:
SE adjustment: Truncated Knapp-Hartung                        tau2 = 1.5e-07
                                                            I2 (%) =    0.00
                                                                H2 =    1.00
                                                   R-squared (%) =  100.00
                                                  Model F(6,4)   =      7.23
                                                  Prob > F       =    0.0381
------------------------------------------------------------------------------
     _meta_es |   Coef.   Std. Err.      t    P>|t|     [95% Conf. Interval]
--------------+---------------------------------------------------------------
Selective MBP |    .168     .041       4.10   0.015        .054       .283
Indicated MBP |   -.153     .264      -0.58   0.592       -.886       .579
USA studies   |    .057     .156       0.37   0.732       -.375       .490
Contact hours |   -.023     .014      -1.62   0.181       -.062       .016
Psychoeducati |       0  (omitted)
Physical exer |    .510     .198       2.57   0.062       -.040      1.061
Other meditat |       0  (omitted)
Other/unclear |    .502     .247       2.03   0.112       -.183      1.188
        _cons |    .010     .204       0.05   0.961       -.558       .579
------------------------------------------------------------------------------
Test of residual homogeneity: Q_res = chi2(4) =  1.30    Prob > Q_res = 0.8616
```

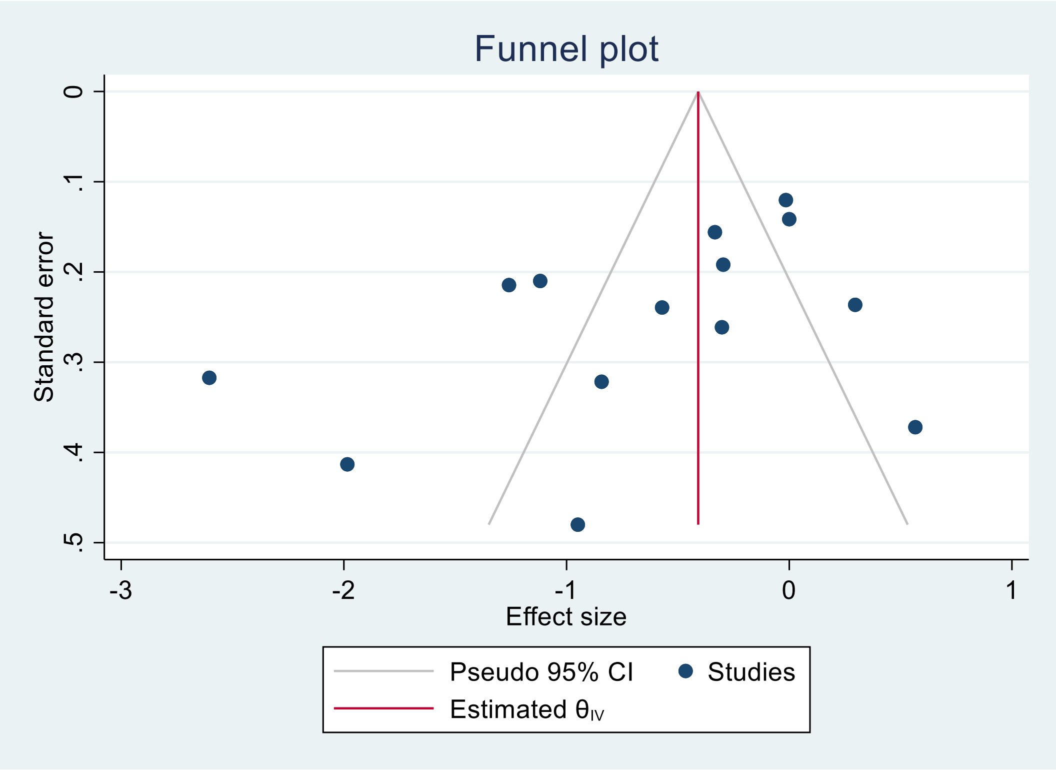

Fig 1. Funnel plot for univariate meta-analysis for the depression outcome at 1-6 months post-intervention for MBPs compared with passive controls.

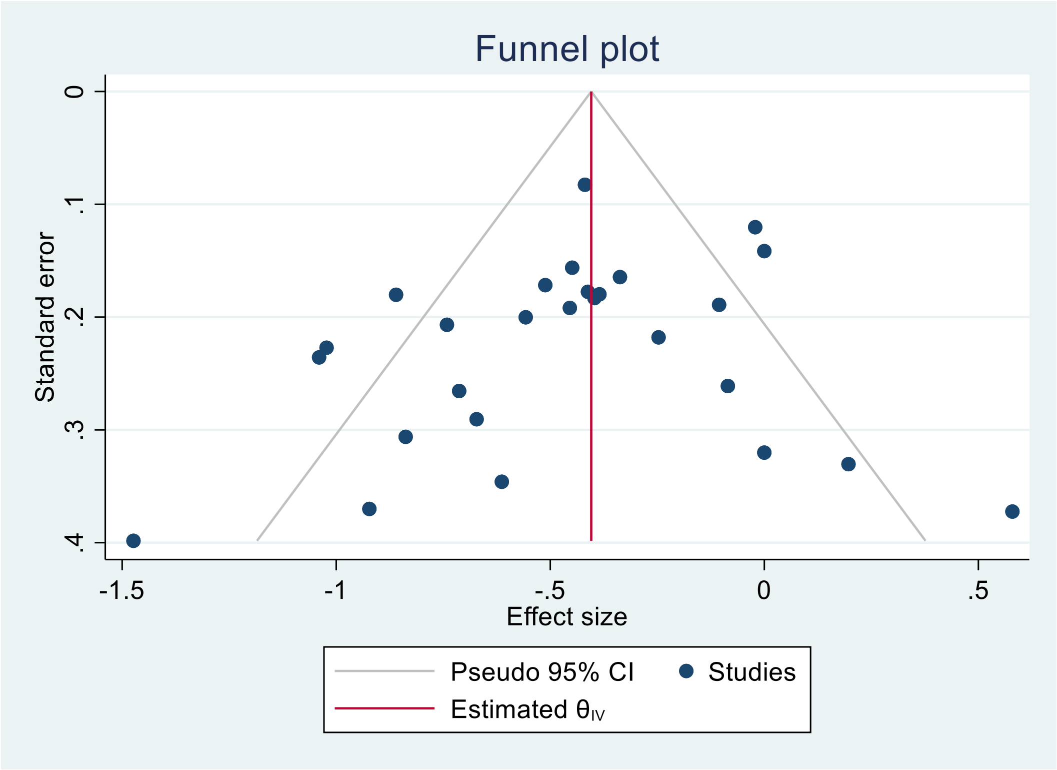

Fig 2. Funnel plot for univariate meta-analysis distress 1-6 months post-intervention compared with passive controls.

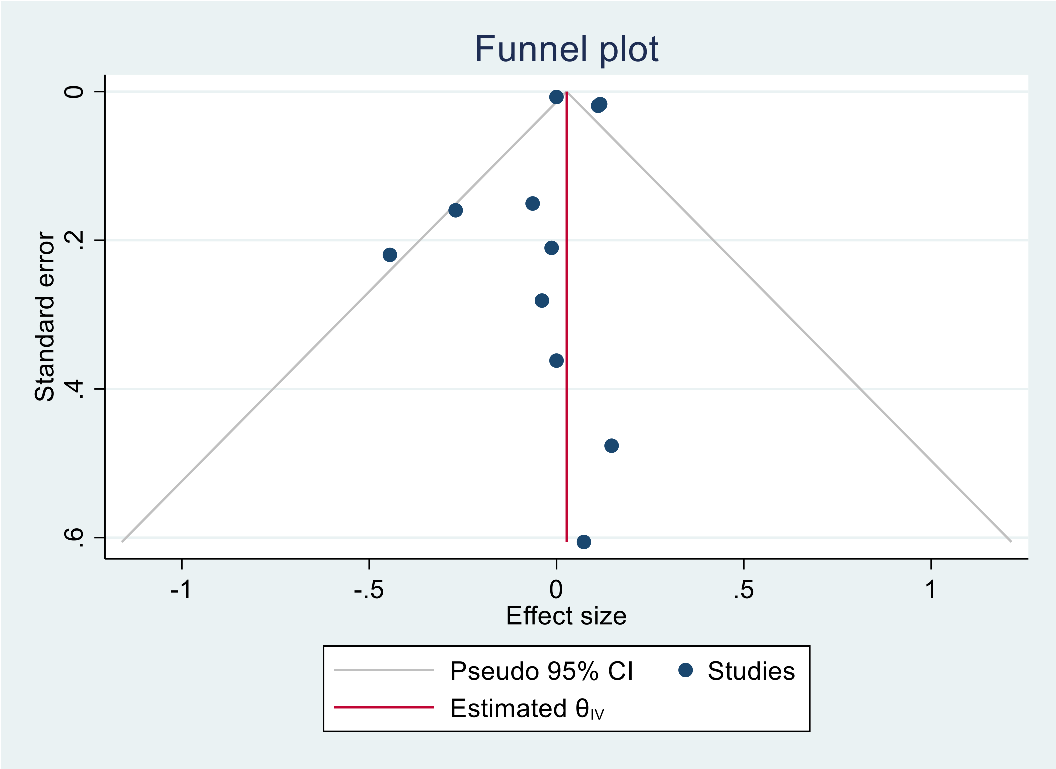

Fig 3. Funnel plot for univariate meta-analysis distress 1-6 months post-intervention compared with active specific controls.
