# Supplementary material for: Mindfulness-based programmes for mental health promotion in adults in nonclinical settings: A systematic review and meta-analysis of randomised controlled trials
Source: PLoS Med. 2021 Jan 11;18(1):e1003481. doi: 10.1371/journal.pmed.1003481 (PMC7799763; doi:10.1371/journal.pmed.1003481)
Supplement: Attachment — Submitted filename: Reply to reviewers v2.docx [file pmed.1003481.s003.docx]

*We wish to sincerely thank the editors and reviewers for their very helpful suggestions to improve our manuscript. Please find our concise point-by-point replies below (line numbers refer to the marked up manuscript).*

# Requests from the editors:

*Please structure your abstract using the PLOS Medicine headings (Background, Methods and Findings, Conclusions - "Methods and Findings" is a single subsection).

*REPLY: We have done this (line 46).*

*In the last sentence of the Abstract Methods and Findings section, please include a brief note about any key limitation(s) of the study's methodology (the authors already note that confidence per GRADE is moderate to very low, this could simply be rephrased slightly to more explicitly state this as a limitation of the SR).

*REPLY: We have rephrased the sentence as advised: “The main limitation of this review is that confidence according to the Grading of Recommendations Assessment, Development and Evaluation (GRADE) approach is moderate to very low, mainly due to inconsistency and high risk of bias in many trials.” (lines 74-77).*

*At this stage, we ask that you include a short, non-technical Author Summary of your research to make findings accessible to a wide audience that includes both scientists and non-scientists. The Author Summary should immediately follow the Abstract in your revised manuscript. This text is subject to editorial change and should be distinct from the scientific abstract. Please see our author guidelines for more information: <https://journals.plos.org/plosmedicine/s/revising-your-manuscript#loc-author-summary>

*REPLY: We have added the Author Summary.*

*In the paper the authors state throughout the writeup that they have conducted multivariate meta-analyses, but at the end of the Methods section the authors use the term multivariable instead; can we just check here that the difference in terminology is intended as of course the two terms do have slightly different meanings and often multivariate is used where multivariable would be the correct term.

*REPLY: Yes, the difference is intended: we have conducted multivariate meta-analyses (i.e. with more than one outcome variable), and multivariable meta-regressions (i.e. with more than one independent variable (regressor)). For clarity, we have reworded the section where the term multivariable is used to: “We used random-effects multivariable meta-regression within multivariate meta-analyses” (lines 292-293).*

*One reviewer has queried the statement in the discussion (Limitations section) that the SR authors have a lack of allegiance with regard to MBPs; the academic editor (AE) backed up this concern. Obviously when the researchers are evaluating whether there was allegiance/lack of allegiance in the trials they've included they have some degree of objectivity/independence (except perhaps where they themselves are triallists), but the same is not true of the SR itself. So the AE felt that this statement could be reconsidered and either justified or rephrased in some way, especially since some authors are triallists in some of the included trials.

REPLY: *Yes, some of us have previously been involved in mindfulness research and conducted one of the included trials. However, none of us has developed or taught any of the mindfulness-based programmes. We have therefore rephrased this as “the fact that none of us has developed or taught any of the included MBPs” (lines 649-650) . In the methods section, risk of bias assessment, we have also added “None of us assessed their own trial” (line 239).*

-----------------------------------------------------------

Comments from the reviewers:

# Reviewer #1:

I confine my remarks to statistical aspects of this paper. The general approach is very well done, but I have a couple comments on it and some suggestions for the tables and figures.

NOTE: It would help to have pages and line numbers

*REPLY:* *We have done this.*

Abstract: The sentence about multiple comparisons is unclear. What exactly was done? Bonferonni? Or something else?

*REPLY: Yes, we applied a conservative Bonferroni correction. We have added this in the abstract: “Multiple testing was performed using p<0.0125 (Bonferroni) for statistical significance” (line 52).*

The fact that the GRADE scores were pretty low will reduce the power to find effects, if they are there, and may increase bias.

*REPLY: We have slightly expanded this mention in the abstract: “Confidence according to the Grading of Recommendations Assessment, Development and Evaluation (GRADE) approach is moderate to very low, mainly due to inconsistency and high risk of bias in many trials.” (lines 76-77).*

The first line of the introduction isn't really meaningful. "Top cause" depends on how causes are listed. E.g. is "mental illness" one thing or many? Instead, give some numbers.

*REPLY: We replaced this with: “With major depression listed as the second largest cause of global years lived with disability, and several other mental disorders within the top 25,…” (line 111).*

Table 2 - I like having as much as posssible of the page devoted to the graph itself. So I would

1. Put the domain under the group, so it is one column, with domain slightly indented

2. Make one column with three rows per study that includes row 1 SMD and p value row 2 CI and row 3 PI

3. Remove the word "confidence"

4. Expand the graph itself

In addition, there should be a "favors control" label and a scale for the axis.

*REPLY: We have done as advised to expand the graph (except point 1 which did not result in gains). We are happy to revise it again if the changes are not agreeable. Please note that this table has been converted into a figure (Fig 2) due to journal requirements.*

Figure 2 Stacked bars are not recommended. Maybe a Cleveland dot plot would be good, or maybe a mosaic plot

*REPLY: This is the standard and widely used Risk of Bias graph format advised by the Cochrane Collaboration. We would prefer to leave it as it is for consistency and to facilitate comparisons with other reviews using the same tool.*

Peter Flom

-----------------------------------------------------------

# Reviewer #2:

This is a pre-registered meta-analysis of 136 trials including 11,605 participants examining the effectiveness of Mindfulness Based Programmes to improve psychological distress and wellbeing in non-clinical populations.

Abstract

For reasons of readability, I'd prefer the authors to refer to the outcome measures in a consistent order throughout the manuscript. There is some variability in how they report this in the abstract and the rest of the manuscript.

*REPLY:* *We have adopted a consistent order.*

I'd refer to "interventions without specific effects or outcomes" as "non-specific active control conditions", as is more commonly done. And "specific effect interventions" as "specific active control conditions".

*REPLY:* *We have followed these suggestions (lines 66 and 70).*

I'd also appreciate some specification as to how results differed according to trial and cultural setting in the abstract.

*REPLY: We have rephrased this to provide more detail in a succinct way: “Only effects on distress remained when higher-risk trials were excluded. USA-based trials reported smaller effects. MBPs targeted at higher-risk populations had larger effects than universal MBPs” (lines 71-74).*

It is not entirely clear at this stage what the authors mean by selective and indicated MBPs, so they might need to clarify that as well.

*REPLY: We have rephrased this: “MBPs targeted at higher-risk populations had larger effects than universal MBPs” (line 74).*

And it might be more helpful if the authors specify in which setting they can expect MBPs to work or not, rather than just mention they cannot be expected to work in every setting.

*REPLY: Other than the information gathered from the moderation analyses described in the replies above, we are unable to tell in which setting they may work or not. This is why we recommend that MBP implementation in non-clinical settings should be partnered with further research to improve targeting. To reflect this more clearly, we have rephrased the last sentence: “Implementation of MBPs in non-clinical settings should be partnered with thorough research to confirm findings and learn which settings are most likely to benefit” (line 82).*

Introduction

It is a strong point of the meta-analysis that it only includes RCTs, that it includes unpublished reports and studies in other languages than English. And that the review procedures were developed with public and professional stakeholders.

For reasons of clarity, I would consider to move effects on secondary outcome measures, such as functioning, relationship with the self and psychosomatic symptoms to the supplementary materials. Or even omit altogether from the manuscript.

*REPLY: We have moved secondary outcome measures to the supplementary materials.*

Methods

The meta-analysis was restricted to group-based first generation MBPs. Particularly with regard to non-clinical populations, online interventions might be both more acceptable and more feasible and are increasingly applied. In this light, I think the authors should comment on their choice in a bit more detail and possibly include this format in their implications for future research or practice.

*REPLY: We have commented on our choice as follows in the methods section: “Online MBPs were excluded as we believe they are different enough from in-person MBPs (e.g. typically not group-based, and fully or semi-automated) to merit their own separate analysis” (lines 181-183). We have also included a related reflection in the discussion/implications section (lines 688-694).*

As the group-based first generation MBPs classically consist of 8 sessions of 2 hours duration, it is slightly puzzling how the authors could have included interventions of only four one-hour sessions. Could the authors elaborate on this?

*REPLY: Several previous trials considered 4 sessions out of 8 a “minimum dose” (e.g. Kuyken, 2008; Teasdale, 2000). On the other hand, many MBPs in non-clinical settings, including some that call themselves MBSR, reduce the length of each session to one hour in order to make it more acceptable for their settings (e.g. offices in Klatt, 2009). Therefore, we considered reasonable to set a minimum of four one-hour sessions or their equivalent. Although we recognise that this was an arbitrary decision, this criterion was pre-specified in the protocol, so the internal validity of the review was not compromised. We now clarify in the methods section: “Studies were deemed eligible if they: (a) were parallel-arm RCTs including cluster-RCTs; (b) assessed group-based first generation MBPs as defined in Crane 2017 (6), with a minimum intensity of four one-hour in-person teacher-led sessions or equivalent (four MBP sessions were used as the “minimum dose” for participants in previous studies (7), and one-hour sessions are common in non-clinical busy settings(8));…” (lines 174-177) . We also added in the limitations: “Some criteria, like including MBPs with a minimum of four hours of instruction, or defining the main outcome time-point range as between 1 and 6 months post-intervention, were reasoned and pre-defined, but ultimately arbitrary limits.” (lines 657-660).*

The authors have chosen to look at assessments between one and six months follow-up. Does this mean that studies only including post-intervention assessments will be excluded from their main analysis? Shouldn't they report in more detail for how many studies this is the case? Although I can follow their line of reasoning, do the costs of this choice not exceed the benefits?

*REPLY: Studies only including post-intervention assessments were indeed excluded from the main analysis, but included in the secondary analyses. The number of studies included in each secondary analysis is reported in Supplementary Table 4 (Table 3 in our first submission). Looking at follow-up data is more clinically relevant because it indicates whether gains are maintained. We slightly expanded our explanation in the methods section to read: “Measures taken less than one month after programme completion may not inform stable changes making them less clinically relevant*“ (line 194). *Also, follow-up assessments may be somewhat less prone to self-reporting bias, and studies assessing follow-up time points are typically larger and better designed. We believe that these benefits outweigh the reduction in statistical power resulting from pooling fewer studies. Although fewer studies measured outcomes 1-6 months post-intervention, we still found several of them, and when pooling them we obtained broadly similar results than with post-intervention data. However, we now recognise in the limitations section that these time-point limits were arbitrary: “Some criteria, like including MBPs with a minimum of four hours of instruction, or defining the main outcome time-point range as between 1 and 6 months post-intervention, were reasoned and pre-defined, but ultimately arbitrary limits.” (lines 657-660).*

The authors consider a trial as being unpublished if enrolment started more than three years before their search date. Given my experience with how long it takes to publish trials like this, is this not a bit too restrictive?

*REPLY: When enrolment had started more than three years before our search date we would contact the authors, and only consider it unpublished if they did not reply satisfactorily. Many explained that their publications were under way or being finalised, and we did not consider these as unpublished. We made this clearer in the text: “We attempted to contact authors if trial enrolment started more than three years before our search date, and deemed a trial as unpublished if authors offered either no outcome reports or an account of their absence (56)” (lines 242-244).*

I'd appreciate if the authors could include a bit more information about which measures were most representative of their primary outcome domains: psychological distress, anxiety, depression and particularly mental wellbeing?

*REPLY: We have added the following information in the Study Characteristics section of the results: “The most common primary outcome measures were: for anxiety the Beck Anxiety Inventory, for depression the Center for Epidemiologic Studies Depression Scale (CES-D), for psychological distress the Perceived Stress Scale, and for mental wellbeing the Positive Affect Schedule (PANAS-P).” (lines 324-327).*

Results

Sixteen trails recruited stressed individuals for whom the MBP was considered an indicated preventative intervention. It is a question whether these individuals should be considered non-clinical or clinical. I think the authors should reflect on this in the discussion section of the manuscript a bit more.

*REPLY: In relation to the limitations of decisions that involved researchers’ judgement and simplification, we added: “The characteristics of the participants in MBPs classed as indicated interventions may overlap with those of participants in clinical settings, although we excluded MBPs which required participants to have a clinical diagnosis” (lines 662-665).*

Intervention for healthcare workers are considered to be selective, those for students universal. What about medical students, during their internships?

*REPLY: As Table 1 shows, we considered interventions for medical interns selective, because their clinical responsibilities typically resemble those of healthcare workers more than those of students who are not interns, placing them at higher risk of mental health deterioration. We have clarified this when we describe study characteristics: “MBPs were selective interventions in 47 trials (35%), targeting groups such as healthcare workers, medical interns, carers, school teachers, and pregnant women.” (line 314).*

Opioid use is mentioned as a secondary outcome measure. Is this compatible with a non-clinical population? Opioid abuse is considered to be a psychiatric disorder.

*REPLY: This refers to opioids prescribed for pregnant women to use right before and during labour. We appreciate that this was not clear, now it reads: “All of the outcome measures were self-reported except for some real-life functioning outcomes such as exams, some psychosomatic outcomes such as peri-labour opioid use…” (line 331).*

The authors mention MBPs improved anxiety, depression psychological distress and mental wellbeing. Please use a consistent order in reporting on outcomes for reasons of readability.

*REPLY:* *We have adopted a consistent order.*

I would consider to move the secondary outcomes to supplementary material (post-intervention and longer term outcomes) or even completely omit from the manuscript (cognitive functioning, relationship with the self, etc.).

*REPLY: We have moved the secondary outcome results to the supplementary materials.*

Risk of bias: One of the obvious difficulties with offering a psychological intervention is allocation concealment. Does this have a disproportionate effect on the assessment of risk of bias? I think it might be worth reflecting on this in the discussion section of the paper and offering some advice on how authors could go about this.

*REPLY: We believe that your comment alludes to the mention of allocation concealment in this sentence: “We noted some concerns about biases arising from the randomisation process for three quarters of the studies, mainly due to the lack of mention of allocation concealment efforts; the remaining quarter are mostly low risk.” We understand that allocation concealment is different from blinding to trial arm: allocation concealment efforts are those to “prevent participants or trial personnel from knowing the forthcoming allocations until after recruitment has been confirmed” (Cochrane Handbook for Systematic Reviews of Interventions, chapter 8 section 8.3). It can always be successfully implemented, regardless of whether participants are blind to assigned interventions during the trial. To avoid further confusion, we now refer to allocation concealment as “allocation sequence concealment” (line 437). We have also added the following in the discussion section: “The modest mindfulness trial quality improvement over time may in part reflect low investment in mental health research (82), and challenges around implementing participant blinding and avoiding outcome self-reporting inherent to behavioural mental health intervention trials.” (lines 700-702).*

I would consider to move the source specific sensitivity analyses to the supplementary materials or remove from the manuscript altogether.

*REPLY: We have moved the source-specific sensitivity analyses to the supplementary materials.*

Did I understand correctly that the number of hours of in-person teaching was associated with a reduction of beneficial effect? This seems to be unexpected, I think the authors should reflect on possible reasons for this in the Discussion section of the paper.

*REPLY: We have added a paragraph discussing this: “Our weak finding that longer courses may be slightly less beneficial was unexpected, although it could be a result of multiple testing and residual confounding. Other reviews assessing course duration have not found this to be an important effect moderator (31, 32). Combined, this evidence suggests that MBP courses do not have to be long to be effective.” (lines 618-621).*

Discussion

With regard to trial quality, the authors mention the importance to improve the quality of mindfulness research. They do not provide any specific advice about which aspects are most important and how to go about improving them. I think they should include these in this paragraph or in the recommendations for future research:

-	Many trials do not report credentials of MBP teachers.

-	Lack of mention of allocation concealment efforts.

-	Missing outcome data.

-	Lack of prospective public protocols.

-	Allegiance effects could only be ruled out in seven studies.

-	Very few studies compared MBPs with active non-specific control groups.

-	Most studies do not mention having measured adverse events or effects.

*REPLY: We added a paragraph on this: “The modest trial quality improvement over time may in part reflect low investment in mental health research [287], and challenges around implementing participant blinding and avoiding outcome self-reporting inherent to behavioural mental health intervention trials. However, it is possible to reduce bias with low-resource measures. Allocation sequence concealment can be done simply, and needs to be reported in publications. Authors could easily encourage participants to complete outcome surveys even where they abandon the MBP, and use these data in intention-to-treat analyses. They could also actively ask participants about any unexpected or unwanted effects. It is crucial for future trialists to prospectively register trial protocols in free public registers where they specify a primary outcome measure and time point, and include a primary outcome data analysis plan. In their publications, authors need to add more intervention and teacher details, even if it has to be in supplementary materials. More resource-intensive improvements include establishing research teams with no allegiance to the intervention, using active control groups (particularly active non-specific control groups), and collecting data beyond self-report. Regarding methodological implications for future reviews, our primary outcome results were sensitive to analytic choices, demonstrating how important it is to publicly pre-specify meta-analyses in detail to avoid outcome-led analytic strategy selection.” (lines 700-715).*

In terms of cultural differences, the USA are more familiar with MBPs which might be an explanation of them being less effective. Is there any information about possible other sources of differences, such as socio-economic background, education, racial background etc?

REPLY: *We did not look at socio-economic background, education or ethnicity as moderators of effect in this meta-analysis because they vary at the participant level. Aggregate data meta-analyses are prone to ecological bias when analysing participant-level moderators. But we do recognise their relevance, hence we are setting up an individual participant data meta-analysis to look at them. We now mention this: “We have not analysed individual-level moderators of effect such as baseline mental health, which optimally require an individual participant data meta-analysis (we are setting up one)” (lines 665-667).*

The authors mention that their preliminary post hoc analysis did not find evidence that good teacher training is influential. I seemed to have missed them reporting this in their results section. Could the authors give more details about this?

*REPLY: We have created a paragraph expanding on this analysis: “We performed a post hoc sensitivity analysis on our primary outcomes excluding trials with unclear teacher competence. There was no change in the size or significance of the estimates, except for the effect on anxiety in comparison with passive controls, which became stronger and with prediction intervals excluding the null (Tables 45-47 in S1 Appendix)” (lines 508-511).*

Under the strengths of this review the authors mentioned their lack of allegiance. However, some of the authors (including the first author) have previously been involved in mindfulness research, and even in one of the included trials, if I have noticed correctly?

*REPLY: Yes, some of us have previously been involved in mindfulness research and conducted one of the included trials. However, none of us has developed or taught any of the mindfulness-based programmes. We have therefore rephrased this as “the fact that none of us has developed or taught any of the included MBPs” (lines 649-650). In the methods section, risk of bias assessment, we have also added “None of us assessed their own trial” (line 239).*

As previously mentioned, I think the recommendations for research (and practice too) are too general. I think the readers would benefit from more specific advice on how to improve both research and the application of MBPs in non-clinical population based on the findings of this meta-analysis.

*REPLY: We have reorganised the discussion section and significantly expanded the Implications for practice and research section (line 668 onwards).*

-----------------------------------------------------------

# Reviewer #3:

The paper addresses an interesting issue in a comprehensive way. It is well written, and the methods are executed impeccably. In fact, their methodological skills in terms of meta-analysis execution seem to be greater than my own, so I will refrain from commenting in this regard.

Some comments:

"Finally, formal meta-analysis of the synthesised data is infrequent and sometimes neglects to disaggregate trials with active versus passive control groups". Although you use "sometimes" maybe you should cite publications that do disaggregate or discard active control groups. For instance, these recent publications: Mindfulness, 10(7), 1193-1216. <https://doi.org/10.1007/s12671-018-1062-5> and The Journal of Positive Psychology, 14(5), 625-640. <https://doi.org/10.1080/17439760.2018.1519588>.

*REPLY: We have added a clarification and included these references: “Finally, formal meta-analysis of the synthesised data is infrequent and sometimes (but not always, e.g. (26), (37)) neglects to disaggregate trials with active versus passive control groups” (lines 144-145).*

Why you did not include common moderators such as year the study was performed, quality of the study, mean age, or gender proportions of the participants?

*REPLY: We performed a sensitivity analysis that considered study quality, excluding trials at highest risk of bias, and confirmed that most results changed after this, showing effectively that study quality is a moderator. We did not look at gender and age as moderators of effect in this meta-analysis because they vary at the participant level. Aggregate data meta-analyses are prone to ecological bias when analysing participant-level moderators. But we do recognise their relevance, hence we are setting up an individual participant data meta-analysis to look at them. We now mention this: “We have not analysed individual-level moderators of effect such as baseline mental health, which optimally require an individual participant data meta-analysis (we are setting up one)” (lines 665-667).*

The section "Implications for practice and research" seems to be underdeveloped. You put forward some strong ideas and you point the way to new avenues of research, but it is unclear what the impact should be in practice. Should Mindfulness practitioners be careful when working with non-clinical populations (as it has been stressed in some clinical populations)? If yes, in which way? If you are discussing the use of Mindfulness as a preventative intervention why do not you bring forward key literature on the topic (e.g. Preventive Medicine Reports, 5, 150-159. <https://doi.org/10.1016/j.pmedr.2016.11.013>)?

*REPLY: We have reorganised the discussion section and significantly expanded the Implications for practice and research section as suggested (line 668 onwards).*

-----------------------------------------------------------

We hope you will find that the manuscript is now acceptable for publication. However, if there are any issues still outstanding, we are happy to review it again.

Thank you,

The authors
