# Supplementary material for: Mindfulness-based programmes for mental health promotion in adults in nonclinical settings: A systematic review and meta-analysis of randomised controlled trials
Source: PLoS Med. 2021 Jan 11;18(1):e1003481. doi: 10.1371/journal.pmed.1003481 (PMC7799763; doi:10.1371/journal.pmed.1003481)
Supplement: Attachment — Submitted filename: Reply to Editors v2.2.docx [file pmed.1003481.s004.docx]

Thank you for your guidance, please find our concise point-by-point replies below.

**Requests from Editors**

1.Title: We suggest revising to: “Mindfulness-based programmes for mental health promotion in adults in non-clinical settings: A systematic review and meta-analysis of randomised controlled trials”

Reply: Done.

2.Data availability statement: At this time your statement reads “All XXX files are available from the XXX database (accession number(s) XXX, XXX.).” Please update the data availability statement with the description of where the data underlying the study may be found.

Reply: Done, we added a permanent DOI. The link is not functional at the moment, but it will become live once we receive formal acceptance and can tell the data repository that the dataset is final. Once we tell them this, we can no longer change the dataset or remove the link from the web, so we need confirmation from you first that the manuscript has been accepted.

3. Abstract: Background: In the last sentence of the Abstract’s Background section, please conclude with a clear description of your study’s main question or objective.

Reply: Done.

4. Abstract: Methods and Findings: How were primary outcomes assessed- anxiety, depression, etc. (for example, the Methods mention that these were psychometrically validated)

Reply: Done.

5. Abstract: Conclusions: Please revise the first sentence to: “Compared with taking no action, MBPs of the included studies promote mental health in non-clinical settings, but given the heterogeneity between studies, the findings do not support generalization of MBP effects across every setting.” (or similar to clarify the reason for your conclusion regarding generalizability across settings).

Reply: Done.

6. Author summary: Why was this study done?” Please revise the second bullet point to: “Many randomised controlled trials (RCTs) tested whether mindfulness courses show benefit, but results are varied and, to our knowledge, there are no reviews combining the data from these studies to show an overall effect.”

Reply: Done.

7. Author Summary: What did the researchers do and find?: Please provide brief mentions of “feel-good practices” as examples for context, and also please combine the third and fourth bullet points: “However, findings from these studies suggest that mindfulness programs are neither better nor worse than other feel-good practices [such as…], and that RCTs in this field tend to be of poor quality, so we cannot be sure that our combined results represent the true effects.”

Reply: Done.

8. Methods (and throughout text) at line 137, please fix the in-text reference call outs (should be [26,37] and please check all the in-text call outs (remove any extra spaces).

Reply: Done.

9. Methods: Line 145-146: Thank you for referencing your published protocol. Please be sure to describe here any instances where the analyses reported here differ from the published protocol and when any such changes were made, including any additional analyses or changes in response to reviewer feedback.

Reply: Done.

10. Methods: Line 148-149: Please include the PRISMA checklist as a supporting information document, and refer to it here (S1_Checklist).

Reply: Done, we completed and added the checklist.

11. Methods: Line 151-152: The search was conducted through January 2020. We request you search be updated to the present time.

Reply: We have updated the search in accordance with the requirement stated in your last email that systematic reviews are current to within six months of anticipated publication date.

12. Methods: Line 225: For clarity, please revise to “None of the authors assessed risk of bias of their own trial”

Reply: Done.

13. Results: In addition to referring to specific tables, please provide the primary outcome results complete with 95% CIs and p values in the text. Where p values are reported, please note p<0.001 where this is applicable.

Reply: Done.

14. Results: Line 335: It would be helpful to refer to briefly list the secondary outcomes together with reference to specific files/tables where the various secondary outcome results are reported (e.g. cognitive functioning at post-intervention (supporting information tables S9-11)).

Reply: Done.

15. Results: Line 337-339: Please report 95% CIs and p values associated with improvements in anxiety, depression, psychological distress and mental wellbeing with MBP vs. passive controls.

Reply: Done.

16. Results: Line 340-343: Please reference where the statistics are reported underlying the finding that in 5% trial settings, MBPs may have no effect on anxiety/depression and possibly result in higher distress and decreased wellbeing measures.

Reply: Done, we added the prediction intervals in the text.

17. Results: Line 350- 356: Please provide the result with p values/confidence intervals associated with the performance of MBPs in comparison to non-specific active control groups.

Reply: Done.

18. Results: Line 359: Please revise to clarify “Too few studies measured anxiety or wellbeing outcomes for MBPs relative to active control interventions, so their results are unreliable”

Reply: Done.

19. Results: Lines 415-421: Please present the statistics, confidence intervals and p values for the results following the exclusion of studies you identified with three or more sources of bias.

Reply: Done.

20. Results: Line 458: Please change p<0.000 to p<0.001

Reply: Done.

21. Discussion: Line 602: If possible please use more specific descriptions than “Eastern and Western cultures”.

Reply: Done.

22. Discussion: Line 576: Please change the heading for this section to read “Strengths and limitations of this review”

Reply: Done.

23.Discussion: Line 594-596: Where you mention your forthcoming IPD meta-analysis, please revise to: “To address this, We plan to conduct an individual patient meta-analysis, which will allow for the analysis of individual-level moderators of effect, such as baseline mental health."

Reply: Done.

24. Discussion: Line 617-618: Please provide a citation for “The field of online MBPs is growing rapidly both in terms of offer and demand, and the COVID-19 pandemic has only accelerated this growth.” or remove the reference to COVID-19.

Reply: Done (reference added).

25.Discussion: Please end the section with a one paragraph conclusion, summarizing the main findings and future implications of the analysis.

Reply: Done.

26.Please remove the sections titled Funding, Declaration of Interests, and Contributors from the text and ensure that this information is accurately entered into the relevant sections of the manuscript submission form.

Reply: Done.

27. References: Please make sure the reference list uses the "Vancouver" style for reference formatting (capitalization and abbreviations of journal titles, for example), and see our website for other reference guidelines: <https://journals.plos.org/plosmedicine/s/submission-guidelines#loc-references>

28.Checklist: Please provide the completed PRISMA checklist (and refer to it in the Methods, e.g.: "This study is reported as per the Preferred Reporting Items for Systematic Reviews and Meta-Analyses (PRISMA) guideline (S1 Checklist)." When completing the checklist, please use section and paragraph numbers, rather than page numbers.

Reply: Done.

29. Table 1. Please provide a brief explanation in the legend as to why standard deviations for age are missing for some studies.

Reply: Done.

**Production Issues**

FIGURES and GRAPHICS:

1. Please ensure that all main figures are referenced in the manuscript as Fig 1, Fig 2, etc. (including capitalization), rather than Figure 1, Figure 2, etc. Please note, however, that the file names themselves must not include the space, i.e. Fig1.tif, Fig2.tif. Please ensure that all main figure files are clearly labeled as such in the File Inventory.

Reply: Done.

METADATA:

1. Please accurately complete the 'Competing Interests' section on our submission form, including any COIs declared by your co-authors. If you have no competing interests to declare, please add "The authors have declared that no competing interests exist". Otherwise please declare all competing interests beginning with the statement "I have read the journal's policy and the authors of this manuscript have the following competing interests:"

Reply: Done.

2. Please provide a detailed Financial Disclosure statement. This is published with the article, therefore should be completed in full sentences and contain the exact wording you wish to be published.

Reply: Done.

1. Please clarify all sources of funding (financial or material support) for your study. List the grants (with grant number) or organizations (with url) that supported your study, including funding received from your institution.

Reply: Done.

2. State the initials, alongside each funding source, of each author to receive each grant.

Reply: Done.

3. State what role the funders took in the study. If the funders had no role in your study, please state: “The funders had no role in study design, data collection and analysis, decision to publish, or preparation of the manuscript.”

Reply: Done.

4. If any authors received a salary from any of your funders, please state which authors and which funders. If you did not receive any funding for this study, please state: “The authors received no specific funding for this work.”

Reply: Done.

3. In accordance with PLOS' data policy, please ensure that your Data Availability Statement in the submission form clearly identifies how readers can access your data. Note that it will be typeset as it is written so please ensure it is in complete sentences and appears as you would like it to in the published version. For details on our data policy, please see <http://journals.plos.org/plosmedicine/s/data-availability>

Reply: Done.

ARTICLE FILE:

1. Please indicate the corresponding author on the title page with an asterisk (*) and include the corresponding author's email address. Only an asterisk and the email address itself are required.

Please format as follows, omitting any other name, address or contact information:

* [mjg231@cam.ac.uk](mailto:mjg231@cam.ac.uk)

Reply: Done.

2. Please remove author degree titles from the byline of your manuscript file.

Reply: Done.

3. Please ensure that all authors have at least one affiliation listed on the title page and that all listed affiliations are attributed to at least one author. Affiliations should be listed in ascending numerical order in the affiliation list and should match what is provided in the Authors Information in the submission form.

Affiliations should include an institution, a city, and a country (no postcode). US affiliations need the state as well, written out in full (with no abbreviations).

Reply: Done, although the submission form does not allow for authors to have second affiliations, so they are stated in the manuscript title page only.

4. Please ensure that all author names are spelled correctly in the manuscript file and the submission form, including the use of a middle name/initials, and any special characters (accents, umlauts, etc.). Please place first/given names before last/family names. We ask that you ensure they match verbatim. Please be aware that correct spelling and formatting of author names in the article file is vital for accurate indexing in PubMed.

Reply: Done.

5. We have noted that you have the following present in your manuscript: Abbreviations list/ Financial Disclosure/ Competing Interests statement/ Author Contributions

We will remove these section from your manuscript file as this information is automatically pulled from the submission form. Please make sure that the information in the submission form is correct.

Reply: Done.

REFERENCES:

1. Please make sure all references are cited in ascending numerical order in the text.

Reply: Done.

SUPPORTING INFORMATION:

1. Please upload your Supporting Information items individually, rather than in a single file, as you refer to them individually in the text. If you would like to retain the single file please rename internal items (e.g. tables) as A, B, C etc. Please also provide a legend for each under the heading Supporting Information. Supporting Information files should be less than 10 MB each. Please edit any citation of internal items within your Supporting Information files to using letters rather than numbers, e.g. “Table A in S1 Text”. Please ensure these changes are made throughout the manuscript and within the Supporting Information files themselves.

Reply: Done. We no longer refer to internal items individually in the text, but we have added a table of contents to the supporting information file to make navigation easy.

2. Please ensure that all Supporting Information files use the following format verbatim throughout the manuscript (including legends and all in-text citations): S1 Fig, S1 Table, S1 Text, S1 Data, etc. Each file type should start at 1 and increase numerically. Please note, however, that the file names themselves must use an underscore rather than a space, i.e. S1_Fig.tif, S1_Table.xlsx, S1_Text.doc, S1_Data.xlsx, S1_PRISMA_Checklist.docx, etc.

Reply: Done.

3. Please ensure that each supporting information file has a legend at the end of the manuscript file, after the Reference list.

Reply: Done.

4. Please note that your submission cannot contain any references to generic "supporting information" files and instead must refer to specific files using our naming convention (S1 Fig, S1 Table, S1 Text, S1 Data, etc.)

Reply: Done.
